# Long-Term Adoption of Televisits in Nursing Homes During the COVID-19 Crisis and Following Up Into the Postpandemic Setting: Mixed Methods Study

**DOI:** 10.2196/55471

**Published:** 2024-06-06

**Authors:** Tobias Martin, Sarah Veldeman, Heidrun Großmann, Paul Fuchs-Frohnhofen, Michael Czaplik, Andreas Follmann

**Affiliations:** 1 Department of Anesthesiology, Faculty of Medicine RWTH Aachen University Aachen Germany; 2 MA&T Sell & Partner GmbH Würselen Germany; 3 Docs In Clouds Telecare GmbH Aachen Germany

**Keywords:** telemedicine, televisits, telehealth, eHealth, electronic health, older adult care, nursing homes, change management, implementation science, technology transfer, innovation transfer, long-term adoption, COVID-19 crisis, postpandemic, coronavirus, digitalization

## Abstract

**Background:**

There is growing evidence that telemedicine can improve the access to and quality of health care for nursing home residents. However, it is still unclear how to best manage and guide the implementation process to ensure long-term adoption, especially in the context of a decline in telemedicine use after the COVID-19 crisis.

**Objective:**

This study aims to identify and address major challenges for the implementation of televisits among residents in a nursing home, their caring nurses, and their treating general practitioners (GPs). It also evaluated the impact of televisits on the nurses’ workload and their nursing practice.

**Methods:**

A telemedical system with integrated medical devices was introduced in 2 nursing homes and their cooperating GP offices in rural Germany. The implementation process was closely monitored from the initial decision to introduce telemedicine in November 2019 to its long-term routine use until March 2023. Regular evaluation was based on a mixed methods approach combining rigorous qualitative approaches with quantitative measurements.

**Results:**

In the first phase during the COVID-19 pandemic, both nursing homes achieved short-term adoption. In the postpandemic phase, an action-oriented approach made it possible to identify barriers and take control actions for long-term adoption. The implementation of asynchronous visits, strong leadership, and sustained training of the nurses were critical elements in achieving long-term implementation in 1 nursing home. The implementation led to enhanced clinical skills, higher professional recognition, and less psychological distress among the nursing staff. Televisits resulted in a modest increase in time demands for the nursing staff compared to organizing in-person home visits with the GPs.

**Conclusions:**

Focusing on health care workflow and change management aspects depending on the individual setting is of utmost importance to achieve successful long-term implementation of telemedicine.

## Introduction

### Background

Population aging is an ongoing trend in industrialized countries and significantly impacts the health care sector [1-3]. Germany currently faces a steep increase in the number of older adult citizens and, concomitantly, in people needing care. Compared to the 2.63 million people needing care in 2013, this figure will increase by approximately 32% to estimated 3.48 million in 2030 [4]. As a consequence, more older adults will receive long-term care in nursing homes (NHs). To avoid future overcrowding of German hospitals and control overall health care costs, limiting hospital admissions (HAs) from NHs will be a major challenge. In fact, these HAs from NHs are frequently avoidable, with potentially avoidable HA cases from NHs accounting for €770 million (US $829 million) in avoidable health care costs in Germany each year [5].

Telemedicine is effective in reducing HAs from NHs and is an attractive modality of care, especially in the context of fewer home visits by general practitioners (GPs), a shortage of geriatricians, and difficulties in accessing health care in rural areas [6,7]. Both synchronous telemedicine, where a physician visits a patient in real time, and asynchronous telemedicine, where information on the patient is entered into a telemedical system and reviewed by a physician at a later time, can be implemented in NHs [8].

Another distinction of telemedical modalities is based on whether patients are accompanied by caregivers or not. Televisits are defined by the authors as videoconferencing among a remote physician, an NH resident, and an on-site caregiver (in this setting, a geriatric nurse) while having access to point-of-care (PoC) diagnostic devices that are integrated into the telemedical system. Televisits enable a structured physical examination and direct patient care, delegated by the remote physician and executed by the caregiver. This is not possible in video consultations, defined as simple videoconferencing between a physician and a patient. In the latter, the examination is limited without access to diagnostic devices, and direct patient care is not possible because there is no caregiver next to the patient.

Telemedicine is a safe modality of care and is noninferior in older adult patients presenting acute medical conditions in cases in which they are accompanied on-site by a nursing caregiver [9]. Several studies evaluating telemedicine for older adults have shown a reduced number of emergency department visits and HAs from NHs [10-22]. Despite these positive effects on the overall level of care and the widespread accessibility of telemedical tools, deployment in primary care and NHs is only progressing slowly.

Even though several implementation frameworks for telemedicine have been proposed in the literature [23,24], a lot of projects transferring telemedical or digital innovation into routine care fail due to poor consideration of change management (CM) aspects [25]. CM can be understood as an organized approach to drive organizational transformation from one current state to a new desired state. The concepts and various models commonly used for business transformation can be applied to health care, where new innovations are also constantly integrated [26-28]. While clinical research mainly focuses on creating evidence for better health-related outcomes, CM aims to ensure long-term adoption of change processes by promoting staff engagement and fostering a culture of continuous improvement. In fact, most of the clinical studies assessing the implementation of new technologies such as telemedicine focus on short-term adoption and technical issues but do not specifically address organizational, cultural, and educational challenges [29-31]. In contrast, the structured approach of CM involves methodically planning and monitoring the entire process to promptly identify and address challenges. This allows for the reduction in resistance to change, ensuring a smooth transition for health care providers and patients and, ultimately, enhancing the quality of care and organizational effectiveness. CM methods are action-oriented approaches focusing on managing specific change processes within one institution by directly addressing challenges. The field of implementation science (IS) must be distinguished from CM applied to health care. While IS also aims to understand implementation and the sustainability of implementation efforts, it takes a broader perspective. In fact, IS aims to create generalizable knowledge about effective implementation strategies of evidence-based health interventions by identifying facilitators and barriers across different contexts and health care settings [32].

Currently, many barriers to the implementation of telemedical tools are known, such as unstable internet connections and other technical issues, insufficient acceptance, privacy and security concerns, poor usability of the systems, a lack of patient support from health care professionals (HCPs), inadequate motivation and training, a shortage of staff, poor planning and engagement, and the fear of misdiagnosis or lack of trust in the technology [31,33-36]. However, implementation guidelines are still missing, and the best practice for implementing telemedicine for ensuring long-term adoption is still unknown. Moreover, there is a gap in research concerning the impact on nursing practice triggered by the organizational implementation of televisits.

### Objectives

This study aimed to identify and address major challenges for long-term implementation of televisits as well as evaluating the impact of televisits on nurses’ workload and their nursing practice.

## Methods

### Setting

This study was conducted in 2 NHs located in 2 different rural areas of the federal state of North Rhine-Westphalia (Germany). Both NHs provide long-term stationary care for older adults, with an average resident age of >85 years. Although they are comparable in size (NH 1 provides care for 93 residents, and NH 2 provides care for 90 residents), they differed in the number of employees within the study period (NH 1 had 64 nurses and nursing assistants for a total of 39.6 full-time equivalents, and NH 2 had 37 nurses and nursing assistants for 32.5 full-time equivalents) and in the number of trainee nurses (14 in NH 1 and 69 in NH 2). There were more women (86% of the employees in NH 1 vs 70% in NH 2, trainees excluded) and older employees (mean age 47.8 years in NH 1 vs 33.3 years in NH 2, trainees excluded) in NH 1 than in NH 2. NH 1 was built in the 1970s and is administered by a foundation managing NH 1, a local ambulatory care service, and 1 facility for assisted living for older adults. NH 2 is run by a local nonprofit organization with a history of  >150 years that provides stationary care in 3 NHs. The organization that runs NH 2 also offers care in all the other relevant fields of older adults’ care (intensive care nursing, ambulatory care, assisted living communities, and palliative care). The regional district of NH 1 faces a steeper decline in the number of physicians than that of NH 2. This is reflected in the average travel time to the nearest GP practice, which was twice as long in the district of NH 1 as in the district of NH 2 (4.4 vs 2.0 minutes) in 2021 [37]. For the medical care of the residents, both NHs collaborate with a coordinating GP, who cares for approximately one-third to half of the total number of residents living in the NH. This is common in Germany, where the remaining residents are followed up on by other GPs located within the catchment area of the NHs. Only the corresponding GPs, and not the GPs of the other 2 NHs, participated in the implementation of televisits. However, an NH resident followed up on by a GP other than the corresponding one could still benefit from a televisit if the coordinating GP was covering for other GPs on holiday or sick leave.

### Timeline

**Figure 1:**
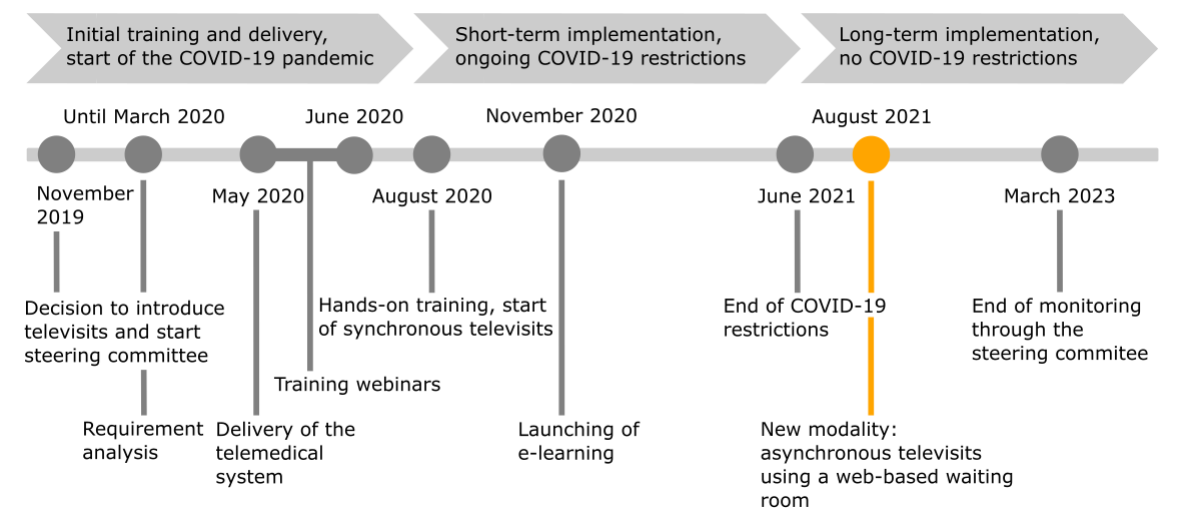
Timeline of the implementation process.

The timeline of the covered implementation process during this study is shown in Figure 1. The total follow-up period between November 2019 and the end of March 2023 can be divided into 3 phases. The first phase between November 2019 and mid-August 2020 consisted in planning the implementation process, supplying and setting up the telemedical system, and initial training of the HCPs. This was followed by 2 distinct implementation phases: a short-term implementation phase until June 2021, during which restrictions due to the COVID-19 pandemic were still in place, and a subsequent long-term implementation phase after the COVID-19 restrictions had been lifted.

From the very beginning, an interdisciplinary steering committee (see the *Evaluation: Steering Committee and Systematic Analysis of the First Implementation Phase* section) organized and supervised the entire change process. After a requirement analysis, the telemedical system was first delivered in May 2020. The initial training was delivered via web-based seminars as COVID-19 restrictions did not allow access to the NHs for external persons. A total of 5 webinars lasting approximately 1 hour each and held consecutively every week covered all the relevant aspects of televisits (the topics were *Introduction to telemedicine*, *Televisits*, *Televisits: the nurse’s perspective*, *Televisits: the GP’s perspective*, and *ECG, auscultation, and other diagnostic devices*). In parallel, the battery lifetime was enhanced, a bigger screen was installed, and the internet connection in the NHs was improved. In August 2020, hands-on training was organized for the entire staff in both NHs. After a short theoretical introduction summarizing the main learning points of the webinars, the participants were trained in televisits using simulated scenarios. Workflow organization was not part of the initial training. The nurse managers in the NHs received advanced training to become superusers, enabling them to feel comfortable in performing televisits, administering the users in the software, and guiding other nurses in their learning process. They later acted as contact persons for their colleagues regarding televisits. After the hands-on training sessions in August 2020, the NHs were asked to perform—whenever possible—weekly televisits with their coordinating GPs while still maintaining the weekly on-site home visits. No specific instructions or guidelines were provided regarding how the televisits should be incorporated into the existing workflows of the NHs. This provided the NHs with the flexibility to integrate televisits according to their specific requirements and organizational contexts.

To allow for continuous training, the webinar sessions, as well as additional step-by-step guides, were made available on an e-learning platform. The nurse managers continued weekly training of their colleagues and accompanied them in televisits when necessary. After a short familiarization period during which the nurse managers provided significant support, effective routine use was achieved between February 2021 and July 2021, covering the last COVID-19 lockdown in Germany from April 2021 to June 2021. When the COVID-19 restrictions ended, the first implementation phase was systematically analyzed within the steering committee. Challenges preventing further implementation were identified and addressed through specific control measures. This led to a new organization of televisits and marked the beginning of a second implementation phase beyond the constraints of the COVID-19 pandemic, referred to in this study as the long-term implementation phase.

The change process was monitored until the end of March 2023.

### Telemedical System

Televisits were performed using the so-called TeleDoc Mobile system (Docs in Clouds TeleCare GmbH), a market-available and mobile medical cart system for televisits with integrated medical diagnostic devices. The TeleDoc Mobile system was equipped with a blood pressure meter (BU 540 connect; medisana GmbH), a blood glucose meter (MediTouch 2; medisana GmbH), a 1-canal electrocardiogram (ECG; WIWE pocket ECG; myWIWE Diagnosztika Kft), and an electronic stethoscope (Littmann stethoscope model 3200; 3M). A conference camera offered a 10-time optical zoom in high definition (PTZ Pro 2; Logitech International S. A.). Screens on both sides of the system allowed for synchronous video communication among the GP, the nurse, and the resident. The software version of the TeleDoc Mobile system underwent multiple updates during the study period from version 1.0 in 2019 to version 3.6.2 in 2023. Feedback from HCPs was directly incorporated into these updates to meet their specific requirements. Previous work on the TeleDoc Mobile system had already demonstrated the technical feasibility of televisits and good acceptance by users [38].

### Evaluation

#### Overview

**Figure 2:**
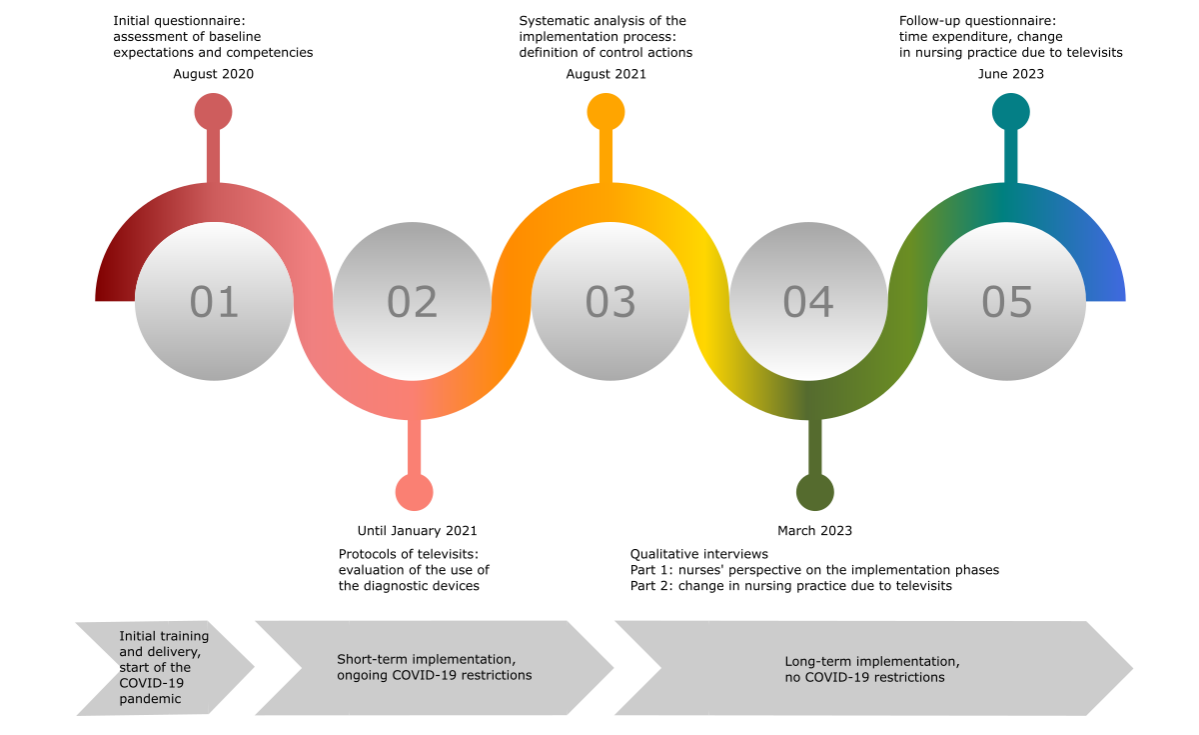
Overview of the methods used for evaluation.

A mixed methods approach was used for evaluation at different time points, as shown in Figure 2. A project diary in paper format containing a questionnaire and televisit documentation protocols (Multimedia Appendix 1) served to assess the nurses’ baseline expectations and competencies and document the first televisits. This diary was distributed to all nurses in the NHs in May 2020, when the final telemedical system was delivered and the NHs were about to be trained and begin but had not yet done real televisits. The nursing staff were asked to fill in the questionnaire and document key elements of their first televisits, such as the date, the motive for consultation, and the number of PoC diagnostic devices used. The diaries were collected in January 2021. As already mentioned, the first implementation phase was observed and systematically analyzed within the steering committee in August 2021. At the conclusion of the study period in March 2023, qualitative interviews were conducted to retrospectively evaluate the nursing perspective regarding the implementation phases and the experienced change related to the implementation of televisits. The qualitative assessment of changes experienced in nursing practice was further quantitatively evaluated using a follow-up questionnaire in June 2023. The individual methods are described in detail in the following subsections.

#### Questionnaire on Baseline Expectations and Competencies

The initial questionnaire (Multimedia Appendix 1) assessed nurses’ self-reported competencies in the use of both medical devices and computer programs. A scale from 1 to 6 following the German school grading system was used (1=very good, 2=good, 3=satisfactory, 4=sufficient, 5=poor, and 6=deficient). Baseline expectations regarding the impact of implementing televisits on the residents’ medical care and the nurses’ time spent on televisits were assessed using preformulated multiple-choice questions. The nurses were also asked to rate, according to the German school grading system and before starting real televisits, their self-perceived level of knowledge of the medical devices as well as their level of understanding of the TeleDoc software. As the questionnaire was linked with the televisit documentation protocols, the questionnaire was not collected directly after the nurses had received training but only with the diaries in January 2021. In total, 20 diaries (n=12, 60% in NH 1 and n=8, 40% in NH 2) were handed back for analysis. After 1 diary was excluded because of missing data, 19 (n=11, 58% in NH 1 and n=8, 42% in NH 2) were used for analysis. Single missing data points were imputed with median imputation. Only the results regarding the self-reported competencies and the initial expectations are presented in the main text of this paper; however, the data for all questions can be found in Multimedia Appendix 2. Continuous variables were described as medians and IQRs and compared using the Mann-Whitney *U* test. Categorical data were described as numbers and percentages and compared using the Fisher exact test. All statistical tests were 2-sided with an α significance level of .05. They were applied in Python (version 3.9.7; Python Software Foundation) using the *scipy.stats* package.

#### Televisit Documentation Protocols

Televisit documentation protocols (Multimedia Appendix 1) were included in the second part of the project diaries. The nurses recorded in these protocols when a televisit took place and whether the televisit was preplanned or organized for an acute issue, such as an acute medical presentation by a resident. The nurses also documented the reason for the consultation and indicated whether they would have contacted the emergency service or the GP out-of-hours service or waited until the GP was available if they had not had access to televisits. In a second part, the nurses were asked to indicate which medical devices they used and rate how well they got along with these devices and the software. Data presentation and statistical analysis were conducted in the same manner as for the data of the initial questionnaire (continuous variables: medians, IQRs, and Mann-Whitney *U* test; categorical variables: numbers, percentages, and Fisher exact test; 2-sided statistical tests; α=.05; Python version 3.9.7). The protocols ended with questions regarding the interaction with the GPs and how the residents felt about the medical care during the televisits. They also contained some open-ended questions for providing personal detailed responses. These data are not presented in this paper but can also be found in Multimedia Appendix 2.

#### Steering Committee and Systematic Analysis of the First Implementation Phase

An interdisciplinary steering committee monitored the entire implementation process. It consisted of members from clinical and change research fields (n=3 physicians doing clinical research and n=2 researchers in CM), a health insurance representative (AOK Rheinland/Hamburg, Düsseldorf, Germany), NH and nurse managers of the participating NHs (n=3), and technical developers from the telemedical system manufacturer (n=2). All authors were part of the steering committee. The steering committee conducted the requirement analysis and organized and developed all forms of training and information material for the nursing staff, such as the hands-on training, the e-learning classes, standard operating procedures for specific presentations, and pocket cards for the use of the medical devices. As the nurses in the NHs reported directly to the senior nurses of their NHs, they were indirectly represented in this committee. Monthly meetings were held throughout the follow-up period to discuss the ongoing process and challenges encountered. If the challenges could not be resolved immediately, specific committee members were assigned to elaborate possible control measures and action plans, which were then presented and discussed in subsequent meetings. The challenges and the resulting actions are presented descriptively. The figures describing this process were created using Inkscape (version 1.3, Free Software Foundation, Inc) and based on free images from Freepik [39] designed by the authors Freepik, Slidesgo (Freepik Company SL), and stories.

#### Qualitative Interviews

The COREQ (Consolidated Criteria for Reporting Qualitative Research) guidelines were used to present the design and results of the qualitative interviews [40]. The COREQ checklist can be found in Multimedia Appendix 3 [40]. From March 2023 to June 2023, a semistructured interview study was conducted with nurses directly involved in the care of the residents (n=5 in NH 1 and n=3 in NH 2) and with senior nurses with additional administrative roles during the implementation of the televisits (n=1 in NH 1 and n=2 in NH 2). These senior nurses had all been part of the steering committee during the implementation process. The interview guide (Multimedia Appendix 4) was developed by TM, a physician and clinical researcher, and checked for consistency and missing questions by HG, a CM expert. The participants were selected through convenience sampling. The nurse managers proposed interview participation to all nurses who had performed televisits. The voluntary participants then received appointments for interviews via videoconferencing. The interviews took place in a dedicated room in the NHs where the nurses were alone and not interrupted during the interview. Before starting the interview, all interview partners were informed about the aim of the interview, which was to collect the individual perceptions and experiences related to the process of implementing televisits in their NH. All interviews were conducted by TM. While the senior nurses with additional administrative roles were known to TM before the interview, there was no previous relationship with the other interviewed nurses. The participants were also informed about TM’s background and that this study on televisit implementation was part of his research for gaining his degree as Doctor of Medicine. They were also informed about his role in the steering committee, where he provided scientific guidance and assistance. The participants received information and gave informed consent for the interviews.

Most of the interview sections were dedicated to evaluating expectations, experiences, and perspectives regarding televisits as well as assessing the impact of their implementation on the nursing practice. These inquiries were all presented as open-ended questions. Another part focused on the addressed challenges and control measures adopted throughout the implementation process. To facilitate focused and structured responses, the nurses were asked to comment freely on predefined statements regarding various aspects of the implementation process within the study period.

The interviews were conducted once without follow-up or subsequent interviews. All the interviews were held in German, visually recorded, transcribed, and analyzed using thematic analysis. No field notes were made during the interviews or when reviewing the interviews. The transcripts were not returned to the participants for comments. As most of the questions directly evoked specific aspects of the implementation process, such as the nurses’ expectations for televisits (asked as follows: “What expectations did you have regarding telemedicine and televisits [note: before the implementation process]?”), the questions themselves predefined the main themes. The coding was done manually within different columns in Microsoft Excel (Microsoft Corp). The responses of the interview partners were divided into sections with different ideas, each collected in separate rows. In a second step, inductive coding was done by defining labels created based on the data. These were then regrouped into categories based on recurring patterns. The labels and the themes were translated into English. To present the data, the different categories under each main theme were condensed into key points or brief sentences.

#### Follow-Up Questionnaire

In June 2023, a follow-up questionnaire (Multimedia Appendix 5) was distributed via a web-based survey tool (UmfrageOnline; enuvo GmbH) to all the nursing staff in NH 1, in which long-term implementation of televisits had been achieved. For comparing pre- and postimplementation results, nurses were tasked with evaluating the time spent on televisits and the impact of implementing televisits on residents’ medical care, mirroring the approach used in the initial questionnaire. In multiple-choice questions, the nurses also reported the impact on nursing practice. Several other aspects, such as the interaction with the GP, the usability of the telemedical system, and the potential of televisits with physicians from medical specialties other than family medicine, were assessed. Only the main results are presented in this paper.

### Ethical Considerations

This study was approved by the ethics committee at the Faculty of Medicine of the Rheinisch-Westfälische Technische Hochschule Aachen (EK 23-178).

## Results

### Questionnaire on Baseline Expectations and Competencies

Before the implementation of televisits, nurses rated both their computer competencies and their clinical skills in taking vital parameters and examining a resident using medical diagnostic devices as good, with slightly better levels in NH 2 (computer competencies: median 2.0, IQR 2.0-3.0, and n=11 in NH 1 vs median 1.5, IQR 1.0-2.0, and n=8 in NH 2, *P*=.06; diagnostic devices: median 2.0, IQR 2.0-2.0, and n=11 in NH 1 vs median 1.5, IQR 1.0-2.0, and n=8 in NH 2, *P*=.10).

**Figure 3:**
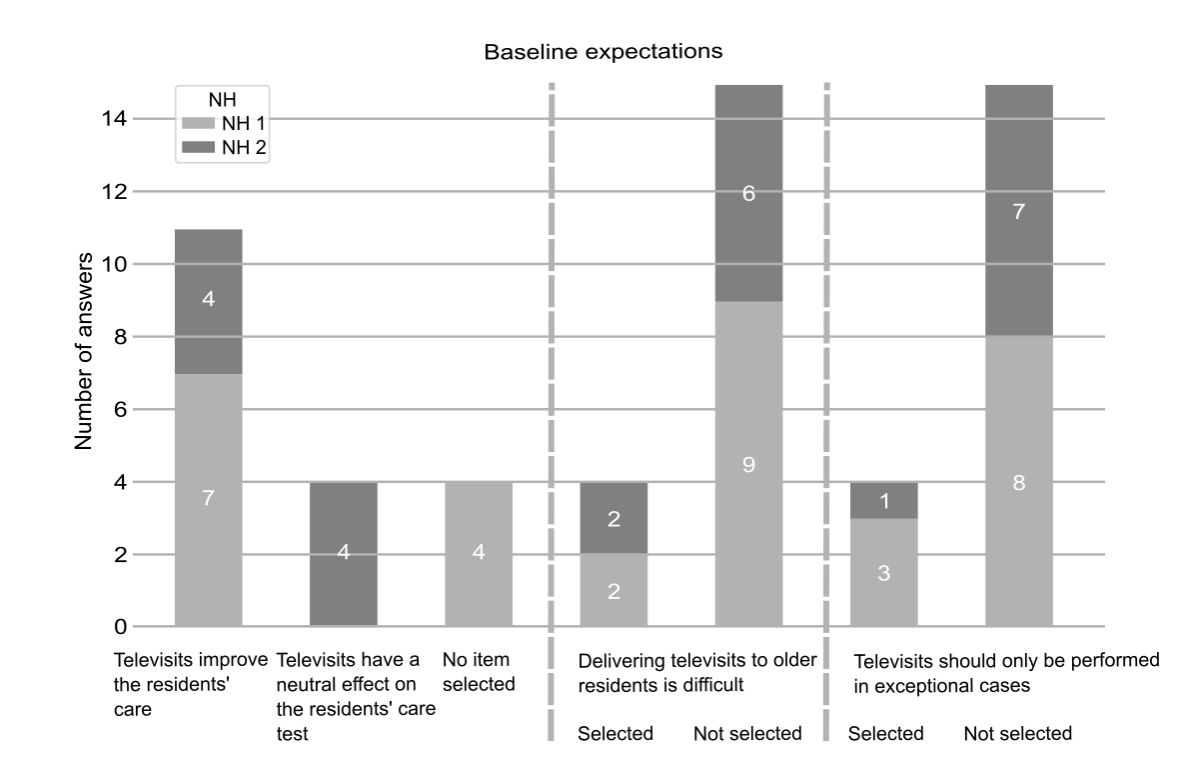
Baseline expectations of the nurses as reported in the initial questionnaire; data presented for n=11 nurses in nursing home (NH) 1 and n=8 nurses in NH 2.

With regard to baseline expectations, slightly more than half (7/11, 64% of respondents in NH 1 and 4/8, 50% of respondents in NH 2; *P*=.66) of the nurses expected televisits to improve the residents’ care, 4 nurses expected the positive and negative effects of televisits to be equal (0/11, 0% in NH 1 and 4/8, 50% in NH 2; *P*=.02), and 20% of the total nurses (4/11 in NH1 and 0/8, 0% in NH2, *P*=.1) agreed with neither of these 2 statements (Figure 3). In total, 4 nurses (3/11, 27% in NH 1 and 1/8, 13% in NH 2; *P*=.60) declared that televisits should only be performed in exceptional cases (see the raw data in Multimedia Appendix 2), 1 (25%) of whom expected that delivering televisits to older residents would be difficult and 2 (50%) of whom did not expect to see positive effects from televisits on the residents’ care (Figure 3). The effect of implemented televisits on the nursing workload was estimated to be neutral by the vast majority in both NHs (9/11, 82% in NH 1 and 6/8, 75% in NH 2; *P*>.99) and to be time saving or time consuming by 3 (1/11, 9% in NH 1 and 2/8, 25% in NH 2) and 1 (1/11, 9% in NH 1 and 0/8, 0% in NH 2) respondents, respectively, with no significant differences between the NHs (*P*=.55 and *P*>.99, respectively).

The initial training sessions on the diagnostic devices were rated as very good to good, and the software instructions were rated as good to satisfactory.

### Televisit Documentation Protocols

A total of 30 televisits in NH 1 and 19 televisits in NH 2 performed between May 2020 and January 2021 were documented by 10 and 6 nurses, respectively, averaging 3.00 and 3.16 televisits per nurse, respectively. Of the total of 49 televisits, 36 (73%; 23/30, 77% in NH 1 and 13/19, 68% in NH 2; *P*=.53) were used for planned routine assessment. In 13 cases (7/30, 23% in NH 1 and 6/19, 32% in NH 2; *P*=.53), the televisits were scheduled at short notice for urgent assessments of residents who had acute medical presentations and issues. In NH 2, all the medical devices provided by the telemedical system were systematically used in every single televisit. In NH 1, the televisits included the use of 1 medical device in 50% (15/30) of cases. In 30% (9/30) of cases, the system was used for videoconferencing without further use of diagnostic devices. In total, 2 or 3 medical devices were only used in 13% (4/30) and 7% (2/30) of televisits, respectively. The blood pressure meter was the PoC device used most often for televisits (16 times), followed by the electronic stethoscope (8 times) and the 1-canal ECG (3 times; Table 1). During these first televisits, the nurses rated their competencies in handling the software and the medical devices as good (Table 2). In an open-ended question asking for possible improvements, they mentioned the need for further training in half (8/16, 50%) of the answers, followed by technological (6/16, 37%) and organizational (2/16, 13%; Multimedia Appendix 6) issues.

**Table 1:** Use of point-of-care (PoC) medical devices in nursing home (NH) 1 within the initial testing and familiarization period. Data presented for a subset of n=21 televisits where one or multiple PoC devices were used. In 9 additional televisits documented in NH 1, no PoC devices were used. The data for NH 2, where measurements were systematically taken using all the PoC devices in every televisit, are not presented (n=19).

Name or number of medical devices			Uses, n (%)
**Medical device**			
	BP^a^ meter	16 (53)	
	Blood glucose meter	2 (7)	
	1-canal ECG^b^	3 (10)	
	Electronic stethoscope	8 (27)	
**Number of devices**			
	No diagnostic devices	9 (30)	
	1 device	15 (50)	
	2 devices	4 (13)	
	3 devices	2 (7)	

^a^BP: blood pressure.

^b^ECG: electrocardiogram.

**Table 2:** Self-reported competencies in handling the telemedical software and the point-of-care medical devices as assessed by the nurses themselves. *P* values are given for the Mann-Whitney U test comparing the results in nursing homes (NHs) 1 and 2. Scale from 1.0 to 6.0, with 1.0 corresponding to very good and 6.0 to deficient.

Point-of-care device and population			Number of ratings, n		Values, median (IQR)		*P* value
**Camera**							
	NH 1	20		1.0 (1.0-2.0)		—^a^	
	NH 2	19		2.0 (2.0-2.0)		.02	
	Total	39		2.0 (1.0-2.0)		—	
**Blood pressure meter**							
	NH 1	14		1.0 (1.0-2.0)		—	
	NH 2	19		2.0 (1.0-2.0)		.73	
	Total	33		2.0 (1.0-2.0)		—	
**Blood glucose meter**							
	NH 1	1		1.0 (1.0-1.0)		—	
	NH 2	19		2.0 (1.0-2.0)		.31	
	Total	20		2.0 (1.0-2.0)		—	
**1-canal ECG^b^**							
	NH 1	3		2.0 (1.5-2.5)		—	
	NH 2	19		2.0 (1.0-2.0)		.47	
	Total	22		2.0 (1.0-2.0)		—	
**Electronic stethoscope**							
	NH 1	8		2.0 (1.75-3.0)		—	
	NH 2	19		2.0 (1.0-2.0)		.09	
	Total	27		2.0 (1.0-2.0)		—	
**Software**							
	NH 1	29		2.0 (1.0-3.0)		—	
	NH 2	19		2.0 (1.0-2.0)		.13	
	Total	48		2.0 (1.0-2.0)		—	

^a^Not applicable.

^b^ECG: electrocardiogram.

### Systematic Analysis of the First Implementation Phase

#### Initial Organization of Televisits

**Figure 4:**
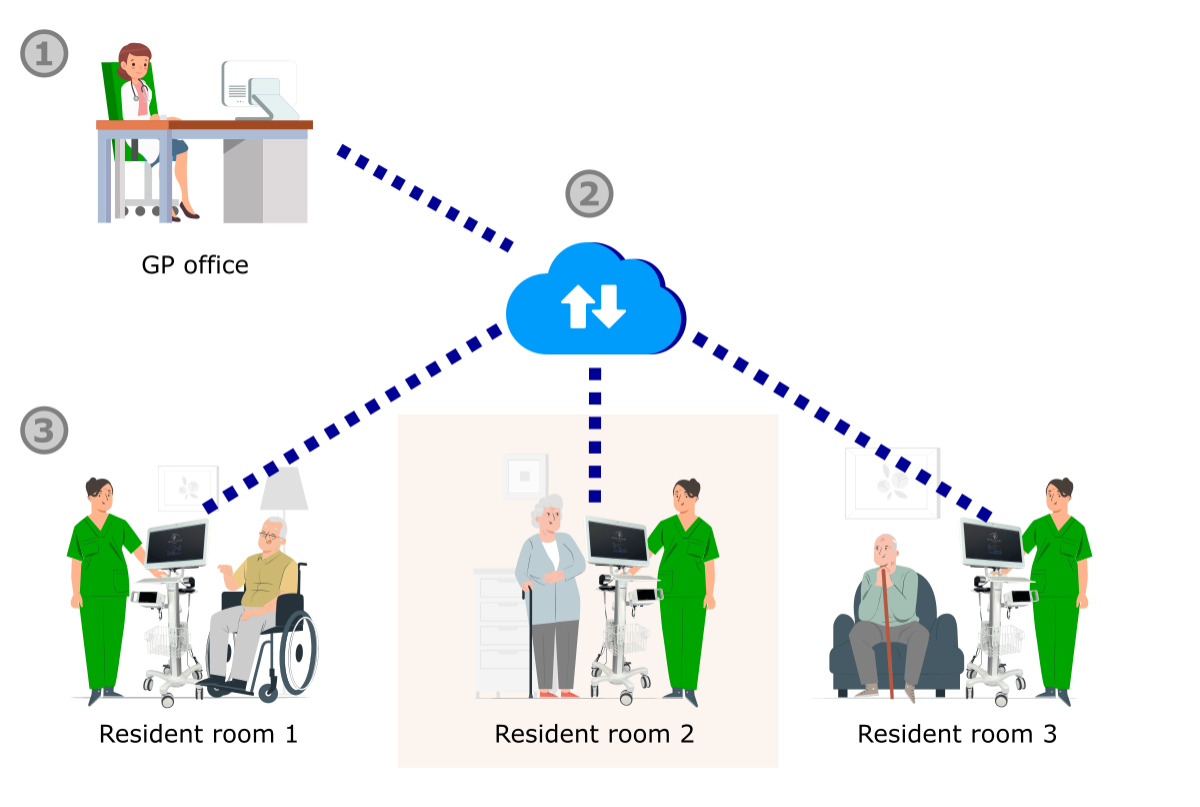
Synchronous televisits in the modality of web-based home visits by a remote general practitioner (GP) in a GP office (1) connected via a telemedical system (2) with a nurse in the nursing home next to a resident (3).

In the first phase of implementation, televisits were organized in the same way as on-site home visits, where a physician sees several residents in an NH while moving between the different residents’ rooms. The remote physician working in the GP office connected via the telemedical system to a nurse in the NH standing next to a resident to be consulted. The televisits had been synchronous at the beginning—thus, the GP in the office as well as the nurse and the resident in the NH both connected to the telemedical interface at the same time and no previous assessment of the resident such as taking of the vital parameters had been conducted beforehand. When the GP requested measurements, the nurses used the medical devices for taking vital signs during the ongoing televisits. Meanwhile, the physician connected to the telemedical system in the GP office via internet waited until completion before deciding which steps to take next. When one televisit had been completed, the nurse on-site moved the telemedical device (with the physician connected via internet) from one resident’s room to another. During these transfer times, the GP also waited. We called this modality *synchronous televisits in the modality of web-based home visits* (Figure 4). During the first implementation period, the issues evoked by the NHs and addressed by the steering committee essentially concerned signal stability, specific software features, and the use of the medical devices. Issues on the side of the GP such as the billing of telemedical visits were also addressed. Televisits were performed regularly in both NHs until the easing of lockdown restrictions in Germany in June 2021.

#### Descriptive Presentation of Encountered Challenges and Adopted Control Measures

##### Health Care Context at the Time of the Systematic Analysis

When the German health care system and society were in transition between the pandemic and the postpandemic situation, the steering committee systematically analyzed in August 2021 the experiences of the first implementation phase. At that point, the last German COVID-19 restrictions were over, and normal home visits were possible again. Resistance to change was rising in the HCPs, who reported a lot of challenges and issues. Three main barriers to long-term adoption were identified: (1) conflicting workflows between the NHs and the GP offices, (2) the lack of time efficiency of the televisits, and (3) perceived uncertainty in using the technology among the nursing staff.

##### Conflicting Workflows

The daily routines and workflows were different in the NHs and in the GP offices. Finding suitable moments for televisits was difficult. For example, noon was a time when there were no consultations in the GP office, and thus, it was ideal for televisits from the GP’s perspective. However, in the NHs, all the staff were busy serving and delivering lunch. Initially, televisits were scheduled at varying times every week, resulting not in the desired flexibility but in organizational stress and discomfort. This was addressed by agreeing on fixed weekly time slots for the routine televisits. With regard to unplanned televisits in the case of acute medical presentations, the workflows were also initially conflicting. The NHs did not bundle calls to the GP office. As a consequence, the workflow of the GP office was interrupted multiple times a day when 2 or more visits had to be planned. During the consultation hours in the GP office, phone calls were particularly disruptive because the GP was seeing other patients at that moment. To avoid disruption due to phone calls, the GP office and the NH grouped requests. Phone calls were completely abandoned except for extreme emergencies. The NH sent a fax containing a list with the residents proposed for consultations along with the reasons for consultation and some contextual information. On the basis of this information, the GP then responded with a fax indicating the vital parameters that should be assessed before the actual consultation (the organization of televisits was changed for an asynchronous modality with vital parameters being taken beforehand; see the following section).

##### Lack of Time Efficiency of the Televisits

**Figure 5:**
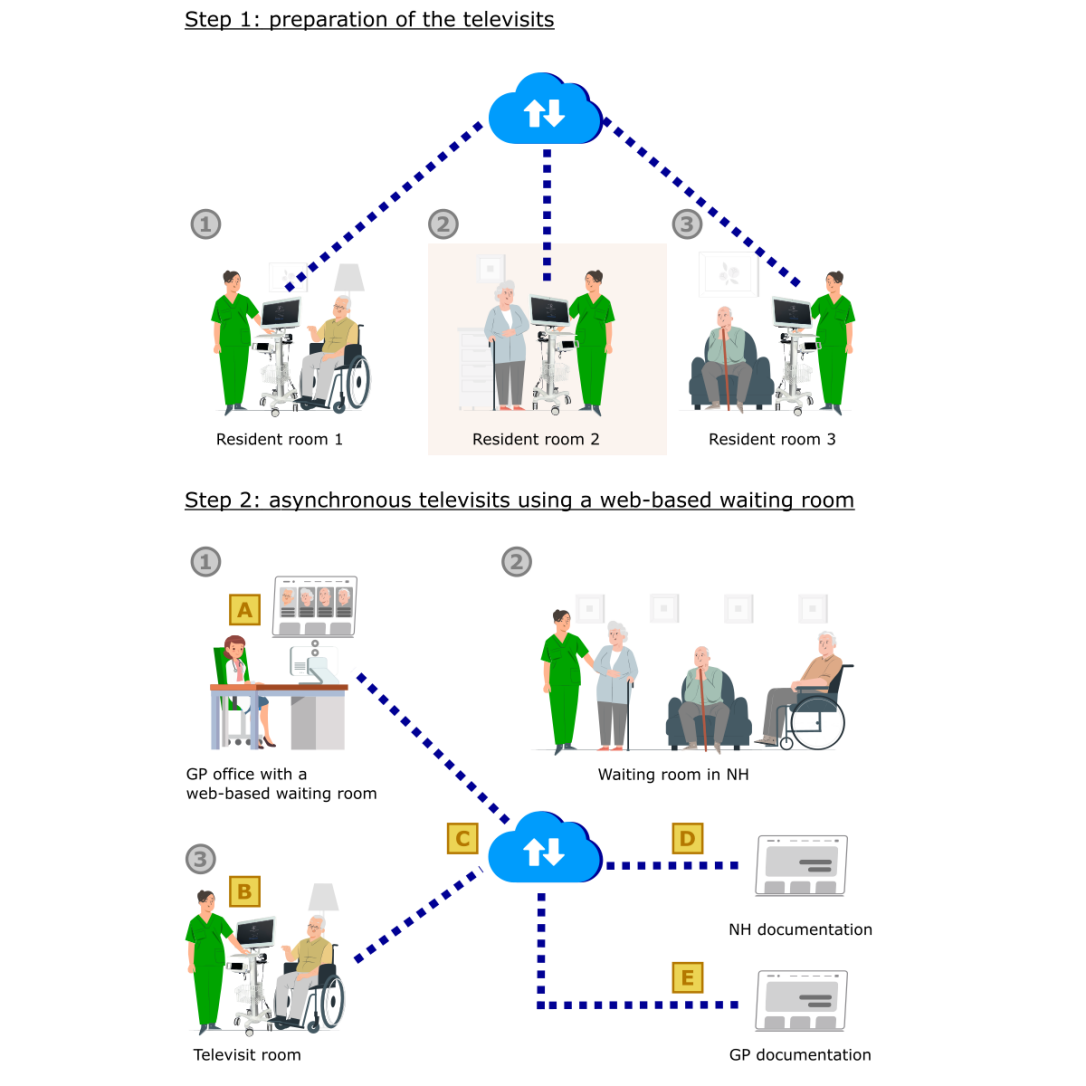
Asynchronous televisits using a web-based waiting room. Step 1: taking of the vital parameters of the residents to be seen. Step 2: asynchronous televisits at a scheduled time using a web-based waiting room for the general practitioner (GP; 1), a physical waiting room in the nursing home (NH; 2), and a defined televisit room (3). The control actions were the creation of the web-based waiting room (A), training of the nursing staff (B), allowing for asynchronous documentation in the software (C), and interfaces to the existing programs (D and E).

The televisits, highly valued during the pandemic phase, were perceived as overly time consuming compared to normal home visits when the latter were regularly possible again. As mentioned previously, the workflows were interrupted during the transit times and when vital signs were taken. This was perceived by the GPs as a significant loss of time they could not spend with other patients in their offices. For the GPs, the time spent on televisits needed to be reduced so that the televisits were a real benefit to them compared to on-site home visits. As these challenges were mainly linked to the synchronous modality of televisits, the organization was completely changed for the modality of *asynchronous televisits using a web-based waiting room* (Figure 5). In this approach, the vital signs of the residents to be seen are taken beforehand by the nurses on-site. Then, with all diagnostic measures completed and documented, the televisits are performed at a scheduled time. The residents to be consulted physically wait in a waiting room in the NH. They also appear in a web-based waiting room in the GP’s telemedical interface. The televisits take place in a dedicated room where the telemedical cart is used while stationary. Instead of moving the telemedical cart, the residents enter the televisit room one at a time as they would a consultation room in a GP office (Figure 5). This greatly improved the time efficiency of the televisits. To avoid losing time spent with doubled-up documentation, interfaces among the telemedical system, the GP, and the NH documentation systems were created. This made it possible to directly export the medical documentation from the TeleDoc software to the other programs.

##### Perceived Uncertainty in Using the Technology Among the Nursing Staff

Moreover, the nursing staff also requested more intense training. To this end, NH 1 and the cooperating GP office jointly hosted a medical student for 4 weeks, during which the student supported the training of the nursing staff. He trained nurses in the use of the PoC devices, helped prepare televisits, and accompanied them during these visits. During these 4 weeks, daily televisits were performed.

Finally, long-term implementation was reached in NH 1, with regular, most of the time weekly, televisits between the GP and NH 1 from August 2021 until the end of follow-up in March 2023. A total of 163 televisits were performed during this period of 20 months. The televisits did not replace the preceding rhythm of on-site home visits but were performed in addition. In NH 2, further efforts to implement televisits after the COVID-19 period remained unsuccessful throughout the follow-up period.

### Interview Findings

#### Selection and Characteristics of Interview Participants

In both NHs, it was planned that the first 5 nurses who volunteered would be included. In NH 2, many nurses considered their experience with telemedicine to be too little and too far away, so no one else wanted to participate after the first 3 nurses were recruited. As NH 2 did not achieve long-term implementation and had not regularly performed televisits since August 2021, additional interviews were unlikely to reveal new themes, insights, or perspectives. Thus, data saturation had already been reached after these 3 interviews. The interviewed nurses were predominantly male (5/8, 62%), with a median age of 26 years. The youngest and oldest participants were aged 26 and 63 years, respectively. The nurse managers were all male (3/3, 100%), with the youngest and oldest participants being aged 30 and 56 years, respectively. The interviews took, on average, 33 minutes for the nurses and 40 minutes for the nurse managers.

#### Interview Findings Concerning the Implementation Phases

In the follow-up interviews, the nurses and nurse managers stated that their main expectation before the implementation of televisits was to enhance residents’ access to physicians, particularly beyond the regular GP consultation hours. The nurse managers further expected a decrease in psychological distress in their staff, along with enhanced legal protection through telemedical expertise during emergency situations with acute health-related deteriorations in their residents.

As regards the subsequent implementation, the interview findings are consistent with the analysis of the steering committee. Nurses in both NHs agreed that televisits were seen as useful during the COVID-19 pandemic and had offered manifest benefits for the residents. Televisits addressed the specific need for thorough medical assessment in the context of the then current contact restrictions. However, the benefits of the televisits seemed less identifiable for most of the nurses of both NHs after the end of the contact restrictions given that normal home visits were possible again and because the telemedical system did not provide the residents with better access to the health care system in terms of other medical specialties or access to physicians outside of the normal office hours. In total, 25% (2/8) of the nurses explicitly saw a benefit of televisits with the GP for residents with acute medical presentations outside the hours of the on-site home visits.

Nurses in NH 1, where long-term implementation was achieved, all agreed that “long-term benefits of televisits became apparent only after many repeated adjustments and training sessions.” The nurses stated that the system became more user-friendly and easier to handle. Repeated use led to more routine use and more self-confidence in the televisits. For this, the repeated training and accompaniment in the televisits were very important for the nurses.

When asked about the factors that had contributed to the success of long-term implementation, the nurses underlined the adoption of asynchronous visits and the transformation of the workflows to a modality with a *web-based waiting room*. They also mentioned the crucial role of the nurse manager’s and the GP’s personal commitment in ensuring the long-term implementation of televisits. The fact that there was a clear benefit for the residents also added to the motivation.

#### Interview Findings Regarding the Change in Nursing Practice Due to Televisits

The 3 interviewed NH and nurse managers and 88% (7/8) of the interviewed nurses retrospectively evaluated the overall experience as positive. However, one nurse felt that the televisits were too impersonal and described difficulties in handling the telemedical system and in performing televisits for people with dementia. This nurse preferred in-person home visits.

The nurse managers stated that televisits reduced the psychological distress experienced by their nursing staff, improved the clinical skills of their employees, and led to greater employee satisfaction during the COVID-19 pandemic (in both NHs) and beyond (for NH 1 in achieving long-term implementation). The interviewed nurses mostly acknowledged the same effect on their clinical skills, but most denied psychological distress and insecurities in their work environment. While most of the nurses stated that asynchronous televisits compared to home visits translated to substantial time savings for the GP, the opinions varied regarding the effect on the nursing workload. Televisits were rather perceived as additional workload when performed for routine visits. In the case of unscheduled visits with GPs, some nurses praised the televisits as time saving, whereas other nurses considered telephone calls to be the faster solution. With regard to consultations outside GP hours, the nurses consistently declared that televisits would allow for a faster medical assessment and reduce nursing workload compared to calling the in-person out-of-hours GP service, for which a considerable waiting time is usual. On top of the organizational and technical challenges regarding the cooperation between the GP offices and the NHs, the nurses described personal challenges in adapting to change, using the technology, and developing effective skills for video communication. Some nurses saw positive effects of televisits on the nurses’ self-image and with regard to legal certainty. Training in simulated televisits and support from nursing peers in a tandem approach were considered adequate to offer continuous training after the initial familiarization phase (Textbox 1).

**Summarized interview findings regarding relevant topics raised during the implementation process.**

**Psychological distress in situations with acute deterioration of a resident’s health status (need for prompt medical assessment)**

- A total of 62% (5/8) of the nurses denied experiencing psychological distress in these situations.
- A total of 38% (3/8) of the nurses described psychological distress in these situations.
- Access to televisits can provide nurses with emotional security and reduce psychological stress.

**Clinical skills of the nurses**

- Most  nurses  saw  improvements  in  their  clinical  skills,  mainly  due  to  more  diagnostic  equipment  being  available  in  the  nursing  home  (NH),  increased  awareness  of  recognizing  changes  in  residents’  conditions,  and  a  more  active  participation  in  the  residents’  medical  care.
- Nurses reported higher confidence in the interactions with physicians since the implementation of televisits.

**Nursing workload**

- Asynchronous televisits saved time for the general practitioner (GP).
- Routine televisits seemed to be associated with a slight increase in workload.
- For  unscheduled  visits,  some  nurses  considered  the  televisits  to  be  time  saving. However,  for  other  nurses,  telephone  calls  seemed  to  be  faster.
- Televisits can be time saving for the nursing staff provided that a telemedical system is always available on standby (no loss of time due to booting), the nurses are experienced in televisits, and a physician is quickly available (direct medical assessment).
- A telemedical service with 24/7 access to a GP was seen as an advantage over the current out-of-hours GP service and would save time for the NHs (reduced waiting time—obsolete telephone waiting line and transfer time of the physician on duty).

**Challenges in performing televisits at the beginning**

- Technical challenges (software bugs, usability, and user experience aspects) and change management (for generating willingness in the nursing staff and the physicians) were seen as challenging at the beginning.
- Collaboration with the physicians, especially in scheduling televisits, and ensuring adequate staffing and support for the televisits were identified as the biggest organizational challenges.

**Communication in a televisit between the nurse, the resident, and the physician**

- It was completely unproblematic for one nurse and difficult and uncomfortable in all televisits for another.
- Most nurses reported challenges in this new setting at the beginning.
- Acoustic problems made televisits difficult for residents with hearing impairments, particularly those who partly relied on lipreading.
- By ensuring a calm and friendly environment and facilitating communication, televisits were possible for residents with dementia.
- Communication is likely to be easier with the next generations of residents in NHs who are already more familiar with modern communication technology.

**Professional identity and self-image of the nurses**

- Nurses understood televisits as a logical and upcoming innovation arriving in the nursing profession in the context of general progress in technology and digitalization.
- However, they did not think that televisits elevate the nursing profession.
- One  nurse  felt  that  his  self-image  in  the  interaction  with  the  physician  improved  by  experiencing  close  cooperation  and  teamwork  in  televisits.

**Medico-legal aspects of the telemedical documentation**

- Main advantage: written prescriptions and medication schedules established by the physician and serving as legal documents are available faster in the NHs.
- However, nurses did not consider the legal certainty that a televisit provides to be higher than that of telephone calls and their subsequent documentation.

**Cooperation between GPs and NHs**

- In both NHs, the cooperation with the physicians was perceived as already good or very good before the introduction of televisits.
- The implementation of televisits did not change the cooperation from the perspective of the nurses.

**Training**

- Training based on a tandem approach, where more experienced colleagues train less experienced colleagues in televisits, was considered useful. Training in simulated televisits with the opportunity to review the use of the technology hands-on meets the needs of the nurses. This also applied for training in the clinical aspects of resident care.

### Follow-Up Questionnaire: Time Expenditure and Change in Nursing Practice Due to the Televisits

The follow-up questionnaire showed that most of the responding nurses performed televisits ≤5 times throughout the study period and only some nurses did so >10 times (>10 times: 4/19, 21%; 5-10 times: 4/19, 21%; 1-5 times: 11/19, 58%; median 1-5 times). Half (9/18, 50%) of the nurses agreed with the statement that televisits were associated with an additional burden related to uncertainty in using the new technology. The nurses rated the time expenditure for performing televisits significantly higher at the end of the follow-up than they did before starting the first televisits. At both timepoints, only very few nurses considered the televisits to be time saving for them (1/11, 9% before implementation, vs 3/18, 17% after implementation; *P*>.99). While the nurses did not expect the televisits to be time consuming in the initial assessment (1/11, 9%), the assessment was significantly different after the implementation process, where the vast majority of the nurses rated them as time consuming (12/18, 67%; *P*=.006). The relative percentage of nurses expecting a neutral effect on the nursing workload was accordingly lower after implementation (3/18, 16%) than before (9/11, 82%; *P*=.001). Compared to the assessment done before the intervention, the evaluation of the impact of televisits on the care of the residents remained unchanged, with half (10/19, 53%) of the nurses estimating a positive effect, one-third (6/19, 31%) estimating a neutral effect, only 1 person estimating a negative effect (1/19, 5%), and two persons with missing answers (2/19, 11%; *P*>.99 for the positive effect and *P*=.71 for the neutral effect when compared to the preimplementation assessment; see the *Evaluation of the Initial Testing and Familiarization Period Up Until January 2021* section). Most nurses fully or generally agreed that televisits were entirely suitable for assessing the residents and for initiating treatment decisions in the case of GP-related medical queries (full approval: 5/19, 26%; predominant approval: 11/19, 58%; mild disagreement: 2/19, 11%; full disagreement: n=0; 1/19, 5% missing answers). As in the interviews, the quantitative evaluation also showed further positive effects of the televisits in addition to improving the clinical and technological competencies of the nurses. These include a sense of empowered participation in the implementation process for about every fourth responding nurse (5/18, 28%), higher professional recognition from the GP and the residents for approximately every third responding nurse (each 7/18, 39%), and a decrease in psychological distress and greater legal certainty for more than half of the respondents (10/18, 56%; Multimedia Appendix 7).

## Discussion

### Principal Findings

#### Key Drivers for Successful Implementation

This study evaluated the implementation of televisits during the COVID-19 crisis in 2 NHs with a follow-up in the postpandemic setting. While both NHs achieved short-term implementation within the pandemic phase, only 1 NH attained long-term implementation. This was achieved in an action-based approach after control measures had been implemented following an analysis of the barriers to further implementation. Adapting the telemedical system and changing the workflows especially to asynchronous televisits with a web-based waiting room were key drivers for successful implementation.

There was no difference in the initial evaluation between the 2 NHs explaining why long-term implementation was only achieved in NH 1. It is noteworthy that, during the first implementation phase, NH 1 adopted a more pragmatic resident-centered approach using only the PoC devices required for the individual patients as shown in the documented televisits. In contrast, NH 2 systematically used all the devices for training purposes in their initial televisits. Moreover, there is a common perception that older nursing staff may possess lower levels of digital literacy, which in turn might render them more resistant to the adoption of new technologies. Interestingly, the staff in NH 1 was older than that in NH 2. Several factors may explain why, contrary to this perception, long-term implementation was achieved in NH 1 with the older nurses.

First, NH 2 simultaneously engaged in a second change process after the end of the COVID-19 pandemic when it changed its NH documentation software in November 2021. At this point, the nursing staff of NH 2 were extremely challenged by adapting to the new software. This competing project was more prioritized than continuing to implement televisits. Second, the interviews revealed that the nurses felt that the personal engagement of the nurse manager and the GP was very important for achieving long-term implementation. This barrier is consistent with findings in the literature indicating that gaining leadership and clarifying roles are important for driving implementation processes [32,34]. Competencies and criteria for leaders in implementing digital health care should be clearly defined in the future to enhance the implementation of telemedicine and digital health [41,42]. Third, NH 1 developed a specific training strategy with the medical student who helped train the nursing staff for 4 weeks. This also contributed greatly to the nurses’ compliance and subsequent implementation.

#### Importance of Considering the User Perspective

The initial vision of how the televisits would be delivered differed significantly from how they were finally integrated by the HCPs to achieve long-term adoption. At the beginning of the transformation process, everybody involved (developers, GPs, nurse managers, and nurses) envisioned a modality that mirrored the workflow of a normal GP home visit (*synchronous televisits in the modality of web-based home visits*). However, another modality, that of “asynchronous televisits using a web-based waiting room,” proved to be more suitable and efficient for routine consultations. The conceptual shift from web-based home visits to the web-based waiting room led to profound organizational modifications and practice changes. Both the participating nurse managers and the developers of the telemedical system stated that they could not have anticipated the web-based waiting room modality before starting the implementation. This highlights the importance of considering the users’ perspective in all phases of development of eHealth solutions, including once a new system is made market available. This approach of broadly involving “a wide range of stakeholders in the entire development process, including especially end-users—patients and physicians” is referred to as “co-creation” [43]. In this study, the HCPs acted as cocreators for developing the asynchronous televisits using a web-based waiting room. They reported their in-use experience with the telemedical system to the steering committee, which triggered discussion on change within the committee. The telemedical system and the workflows were then adapted to allow for asynchronous televisits. This resulted in an improved telemedical system and, thus, a more valuable product. As it provides greater usability for the HCPs, their acceptance of the system and the implementation process increases. The CM approach in this study allowed for this interaction between end users and developers and additionally provided an organized platform for identifying ongoing challenges and steering the transformation process in the right direction. Imposing overly rigid application frameworks for new digital innovation in health care may be a major contributor to the failure of health innovations to be transferred into routine care.

#### Change in Nursing Practice Due to Televisits

In NH 1, the nurses were positive about the long-term implementation and acknowledged improvements in their clinical skills and technological competencies. In addition, the televisits improved their self-image and their recognition as nurses. These positive results were found in the interviews and confirmed in the follow-up questionnaire. When asked directly in the interviews, only a minority of the nurses reported experiencing physical distress when residents show acute medical presentations requiring prompt medical assessment. However, most agreed in the quantitative analysis that access to televisits was perceived as relieving and reducing emotional distress. The lack of consistency here is probably due to response bias, with nurses responding more honestly in an anonymous questionnaire than in a face-to-face interview.

Televisits were perceived as time saving for the GP but were associated with an additional technological burden and a slight increase in time spent by the nursing staff. The latter was not expected by the nurses when they were assessed before the implementation process. Interestingly, this observation is in line with those of other studies [44-46], which also show that televisits in NHs are associated with an increased workload for the nursing staff. Although most nurses in this study expected a neutral effect on workload rather than an increase, the staff in NH 1 adapted to televisits even though they increased their workload. This is a good indication that the other effects are seen as positive and valuable.

The taking of vital parameters using medical diagnostic devices or recording on an ECG are not among the normal tasks of a geriatric nurse in Germany. This means that there is unlikely to be an increase in the overall workload but rather a shift from GP activity to tasks performed by geriatric nurses. The GPs can use the time they save for other patients. This can be beneficial in the context of a shortage of GPs, especially in rural areas. Given the overall positive effects, health insurers and public health managers should consider providing financial support for the implementation of televisits in NHs. In particular, the initial setup and training should be financially supported to maintain high motivation for further implementation.

This work logically builds on and complements the group’s previous research, which demonstrated the technical feasibility and usefulness of integrating PoC diagnostic devices into video consultations for the assessment of older adult patients in NHs [38]. This study shows that there is no need to take all the vital parameters of the residents in every consultation. In more than three-quarters of the televisits (24/30, 80%), no or one single measurement was sufficient. Access to the right diagnostic device at the right time for the right resident with the right physician connected via telemedicine is essential for personalized care and to avoid unnecessary HAs. In line with the idea of effective workforce management, it does not make sense to ask nurses to take all the vital parameters but rather to decide in advance (ie, when planning the televisit) which vital parameters should be taken.

#### Limitations and Future Research

This study has certain limitations for the interpretation and generalizability of the results. The implementation of televisits in NHs was only evaluated in 2 NHs, and 1 failed to achieve long-term implementation. Therefore, generalization to other NHs may depend strongly on their individual organization and health care environment. However, this study shows that an action-based CM approach piloted by an interdisciplinary steering committee can allow for successful implementation. Future studies in the field of IS may add valuable insights to determine more objective criteria for the success of telemedical implementation efforts. Another major limitation is the relatively small number of televisits in the documentation protocols. However, this was sufficient to show that it is not useful to systematically take measurements using all diagnostic devices integrated into the system. This study was not designed to assess how the residents felt about being involved in promoting telecare. However, this is an important research question that should be evaluated in other studies. Furthermore, this study investigated the implementation of one technology—televisits—in NHs. Other digital technologies such as NH managing software, wearables, and home automation systems may also improve workforce management, enhance the quality of care, and provide a better living environment in NHs. As some authors believe that all these technologies are likely to be integrated into so-called “smart NHs,” possibly replacing conventional NHs in the decades to come [47-49], the implementation processes of these technologies should be specifically investigated as generalizability from this study is very limited.

Future research should focus on training and staffing concepts for nursing. First, performing televisits is a new approach to care and needs specific education. While the need for specific curricular training in telemedicine has already been identified for medical students [50-53], it should also be integrated into nursing education. Second, a notable finding in the follow-up questionnaire was that most televisits were conducted by a small number of nurses with some performing a significant number whereas most only conducted a few (>10 televisits: 4/19, 21%; <10 televisits: 15/19, 79%), Therefore, future research should explore whether certain nurses should specialize in televisits within a nursing facility whereas less qualified nurses or nursing assistants could focus on other nursing tasks. Maybe this would lead to workforce optimization. In theory, telemedically advanced nurses could also use telemedicine for telenursing, that is, for advising and supporting nursing assistants via telemedical solutions.

### Conclusions

An action-oriented CM approach made it possible to achieve long-term implementation of televisits in NH 1. Abandoning synchronous televisits in favor of asynchronous ones improved the workflows and was a critical facilitator of long-term implementation. Strong leadership, as well as sustained training of the nurses, also contributed to this success. The implementation of televisits had positive effects on the HCPs. They experienced an improvement in their clinical skills and a higher professional recognition. According to the nurse managers, their psychological distress also decreased. Performing televisits did not save time for the nursing staff in comparison to scheduling and assisting a GP in in-person home visits. Instead, the nurses believed that televisits led to a slight increase in their time spent on organizing the assessment of the patient. CM aspects must be considered to achieve long-term implementation.

## Appendix

Multimedia Appendix 1 Project diary.

Multimedia Appendix 2 Excel (Microsoft Corp) sheet data from project diaries.

Multimedia Appendix 3 COREQ (Consolidated Criteria for Reporting Qualitative Research) reporting guidelines.

Multimedia Appendix 4 Interview guide.

Multimedia Appendix 5 Follow-up questionnaire.

Multimedia Appendix 6 Comments of the nurses regarding improvement potential for further televisits. The number of times a specific topic was mentioned is indicated in brackets.

Multimedia Appendix 7 Nurses’ self-assessment of the positive effects of implementing televisits in nursing home 1. Agreement with the individual aspects shown for 18 nurses; n=1 respondent in the postevaluation period did not select an answer.

## Abbreviations

CM: change management
COREQ: Consolidated Criteria for Reporting Qualitative Research
ECG: electrocardiogram
GP: general practitioner
HA: hospital admission
HCP: health care professional
IS: implementation science
NH: nursing home
PoC: point-of-care
