# Supplementary material for: Long-Term Adoption of Televisits in Nursing Homes During the COVID-19 Crisis and Following Up Into the Postpandemic Setting: Mixed Methods Study
Source: JMIR Aging. 2024 Jun 6;7:e55471. doi: 10.2196/55471 (PMC11190630; doi:10.2196/55471)
Supplement: Multimedia Appendix 1 [file aging_v7i1e55471_app1.pdf]

Logo of a project partner

**This is the version translated into English, the original German version is below (page 29 and following)!**

# Project Diary

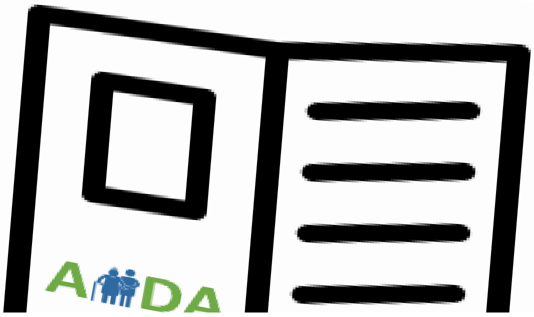

## Version 1

***Note for the nursing home:***

*Please fill in the person code before handing the project diary to the nurse!*

| Code | 1 | 1 | | |
|---|---|---|---|---|

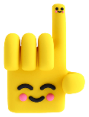

***Note:***

*Please cut off the bottom corner with your name before returning it.*

**This project diary belongs to:**

## Project & diary – At a glance!

### Objective:

The AIDA project aims to develop a practical concept for implementing televisits in nursing homes.

### Project realisation:

To this end, users in the nursing homes and GP offices already use the system in the development phase.

### Method:

This project diary allows systematic recording of expectations and experiences of the users.

### Structure:

The project diary is made up of different sections with different colors.

**Your contact person for questions relative to the project diary:**

**XXXXXXXXXX**
Phone: xxxxxxxxxxxxxxxxx
mailofcontactperson@xxxxxx

**Basic personal data:**
**Information on gender, age, professional experience, etc. is only collected once.**

**General information: This section only needs to be completed at the outset and may need to be revised when new training courses or additional instruction manuals are provided.**

**Protocols for each televisit:**
**The protocol should be completed as soon as possible after each televisit.**

**We would also like to record – with your support – how the residents think about the medical care during the televisits! Please ask them about this and fill in the information at the end of the protocols. You find the question to the residents on the last page of the project diary.**

### Instructions for use:

**Pictograms and symbols are used for visualisation:**

**This symbol refers to specific instructions.**

O **Circles are used to tick relevant items.**

☐ **Rectangles are used for entering digits (e.g. times, ratings)**

☺ **The German school grades you are familiar with are used for the ratings.**

☺ ☹︎ (😐) ☹

| 1 | 2 | 3 | 4 | 5 | 6 |
|---|---|---|---|---|---|
| very good | good | satisfactory | sufficient | poor | deficient |

**If possible, use a felt-tip or ballpoint pen to complete the project diary, please do not use a pencil.**

# Basic personal data

**Please provide us with information on the following points.**

**I am ...** O **female** O **male** **I am... [ ] years old.**

**I have [ ] years of professional working experience** (without training periods) **in nursing.**

**I am...**

O nursing assistant

O registered nurse

O (additionnaly) qualified in something else, namely :

O nursing manager

O nursing director

**Regarding the implementation of televisits in our nursing, I am responsible for special tasks** (z.B. briefing of employees, performing technical updates).

O **yes, for the following tasks: ........................................................................**

**How do you rate your clinical skills in using medical devices, that are, <u>that are already used in your nursing home?</u> Please assign a school grade.**

☺ 😐 ☹

| 1 | 2 | 3 | 4 | 5 | 6 |
|---|---|---|---|---|---|
| very good | good | satisfactory | sufficient | poor | deficient |

[ ]

**How do you rate your clinical skills in using computer programs (e.g. care documentation program)? Please assign a school grade.** [ ]

**What are your expectations regarding the effect of implementing televisits on the medical care provided to the resident? (tick all the statements you agree with)**

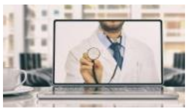

O This significantly improves medical care.

O The positive and negative aspects will be about equal.

O Delivering televisits to our residents will be difficult.

O Televisits should only be performed in exceptional cases.

**What are your expectations regarding the effect of televisits on your nursing prac-tice? Assessing health-related problems of the residents will probably be...**

O be significantly less time-consuming (timesaving for the nurses)

O different, but take about the same time

O time-consuming

**?** Please tick the statement you agree with!

# Information – relevant to the TeleDoc software

**Please indicate the elements you have received information on. Please tick the corresponding circles. Please answer some additional questions for <u>these</u> points.**

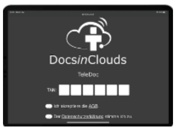


**I have received information about the TeleDoc software.**

O **yes**
O **no**

**I have received information via ...** (multiple answers are possible!)

| | |
|---|---|
| O **an on-site briefing on a computer** | O by project staff<br>O by nursing home staff |
| O **a digital briefing on a computer** | O by project staff<br>O by nursing home staff |
| O **digital training courses/ digital media** | O videos<br>O online presentations |
| O **written information** | O a manual<br>O other documents, namely<br>……………………………………………… |

**How well informed do you currently feel about the TeleDoc software? Please assign a school grade.** ☐

| day | | month | |
|---|---|---|---|
| | | | |

| 1 | 2 | 3 | 4 | 5 | 6 |
|---|---|---|---|---|---|
| very good | good | satisfactory | sufficient | poor | deficient |

**What was difficult to understand in the information on the TeleDoc software or was not covered enough? What would you like to see?**

# Information –point-of-care diagnostic devices

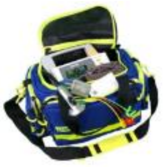

**I have received information about the point-of-care diagnostic devices integrated into the TeleDoc.**

O yes
O no

**I have received information via …** (multiple answers are possible!)

| | |
|---|---|
| O **an on-site briefing** | O by project staff<br>O by nursing home staff |
| O **digital training courses/ digital media** | O videos<br>O online presentations |
| O **written information** | O a manual<br>O other documents, namely<br>………………………………………………………… |

| Which TeleDoc devices are available? | Which devices have you received information on. | Rate the information for every device with a school grade. | | A short guide with visualisations would be good for: |
|---|---|---|---|---|
| O camera | O camera | camera | | O |
| O stethoscope | O stethoscope | stethoscope | | O |
| O blood glucose meter | O blood glucose meter | blood glucose meter | | O |
| O blood pressure meter | O blood pressure meter | blood pressure meter | | O |
| O one-canal ECG | O one-canal ECG | one-canal ECG | | O |
| O spirometer | O spirometer | spirometer | | O |
| O sonography | O sonography | sonography | | O |
| O patient monitor | O patient monitor | Patient monitor | | O namely for |

(with ECG, T°-, BP-, glucose- meter, heart rate and photo-pletysmography)

…………………………………………………………………

**What was difficult to understand in the information on the point-of-care diagnostic devices or was not covered enough? What would you like to see?**

***Congratulations! The survey is now completed!*** 😊 **You will now find *10 protocols for documenting televisits! Please contact us if you need more!***

# Televisit documentation protocol (1)

## Basic information on the televisit

When was the televisit performed?

| day | | month | |
|---|---|---|---|
| | | | |

The televisit ...

- O ... was planned with the GP beforehand.
- O ... necessary for an acute issue (non-planned)

Based on what situation or complaint was the televisit performed?

..............................................................................................................................................

What would you have done in this specific situation if a televisit had not been available?

- O dialed the emergency service via the 112
- O dialed the GP out-of-hours service
- O waited, until our GP was available

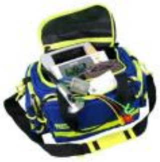

## Use and rating of the integrated point-of-care medical devices

During the televisit ...

- O <u>no</u> medical devices
- O medical devices

were used.

| Which medical devices integrated into the TeleDoc were used? | How well did you get on with the <u>used</u> devices? Please assign a school grade. | |
|---|---|---|
| O camera | camera | |
| O stethoscope | stethoscope | |
| O blood glucose meter | blood glucose meter | |
| O blood pressure meter | blood pressure meter | |
| O one-canal ECG | one-canal ECG | |
| Patient monitor (PM) | Patient monitor (PM) | |
| O ECG via PM | - ECG via PM | |
| O heart rate via PM | - heart rate via PM | |
| O blood pressure via PM | - blood pressure via PM | |
| O blood oxygen saturation via PM | - blood oxygen saturation via PM | |
| O Temperature via PM | - Temperature via PM | |

What was difficult when using the TeleDoc devices? What would you like to see?

*If there is not enough space, use the blank page at the end for further comments.*

# Televisit documentation protocol (1)

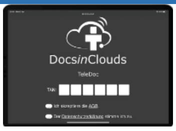


## Rating of the TeleDoc software

How well did you get on with the software?
Provide a school grade from 1 very good to 6 deficient.

What was difficult when using the TeleDoc software? What would you like to see?

## Assessment of the supervision by the GPs

How do you rate the supervision provided by the GPs during the performed televisit?
Please assign a school grade.

| 1 | 2 | 3 | 4 | 5 | 6 |
|---|---|---|---|---|---|
| very good | good | satisfactory | sufficient | poor | deficient |

What would be necessary or desirable to improve the supervision provided by the GPs during this specific televisit?

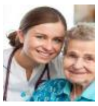

How did the resident feel about the medical care he/ she received during the televisit?
Which school grade did he/she assign for the medical care?

Please enter any comments or remarks from the residents.

# Televisit documentation protocol (2)

## Basic information on the televisit

When was the televisit performed?

| day | | month | |
|---|---|---|---|
| | | | |

The televisit …

O … was planned with the GP beforehand.
O … necessary for an acute issue (non-planned)

**Based on what situation or complaint was the televisit performed?**

..............................................................................................................................

**What would you have done in this specific situation if a televisit had not been available?**

O dialed the emergency service via the 112
O dialed the GP out-of-hours service
O waited, until out GP was available

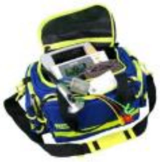

## Use and rating of the integrated point-of-care medical devices

During the televisit …

O <u>no</u> medical devices
O medical devices

were used.

| Which medical devices integrated into the TeleDoc were used? | How well did you get on with the <u>used</u> devices? Please assign a school grade. | |
|---|---|---|
| O camera | camera | |
| O stethoscope | stethoscope | |
| O blood glucose meter | blood glucose meter | |
| O blood pressure meter | blood pressure meter | |
| O one-canal ECG | one-canal ECG | |
| Patient monitor (PM) | Patient monitor (PM) | |
| O ECG via PM | - ECG via PM | |
| O heart rate via PM | - heart rate via PM | |
| O blood pressure via PM | - blood pressure via PM | |
| O blood oxygen saturation via PM | - blood oxygen saturation via PM | |
| O Temperature via PM | - Temperature via PM | |

**What was difficult when using the TeleDoc devices? What would you like to see?**

*If there is not enough space, use the blank page at the end for further comments.*

# Televisit documentation protocol (2)

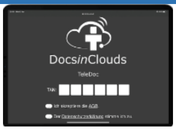


## Rating of the TeleDoc software

How well did you get on with the software?
Provide a school grade from 1 very good to 6 deficient.

What was difficult when using the TeleDoc software? What would you like to see?

## Assessment of the supervision by the GPs

How do you rate the supervision provided by the GPs during the performed televisit?
Please assign a school grade.

| 1 | 2 | 3 | 4 | 5 | 6 |
|---|---|---|---|---|---|
| very good | good | satisfactory | sufficient | poor | deficient |

What would be necessary or desirable to improve the supervision provided by the GPs during this specific televisit?

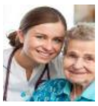

How did the resident feel about the medical care he/ she received during the televisit?
Which school grade did he/she assign for the medical care?

Please enter any comments or remarks from the residents.

# Televisit documentation protocol (3)

## Basic information on the televisit

| When was the televisit performed? | day | | month | |
|---|---|---|---|---|
| | | | | |

The televisit …

- O … was planned with the GP beforehand.
- O … necessary for an acute issue (non-planned)

**Based on what situation or complaint was the televisit performed?**

…………………………………………………………………………………………………………………

**What would you have done in this specific situation if a televisit had not been available?**

- O dialed the emergency service via the 112
- O dialed the GP out-of-hours service
- O waited, until out GP was available

## Use and rating of the integrated point-of-care medical devices

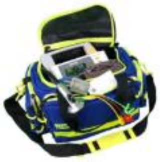

**During the televisit …**

- O <u>no</u> medical devices
- O medical devices

were used.

| Which medical devices integrated into the TeleDoc were used? | How well did you get on with the <u>used</u> devices? Please assign a school grade. | |
|---|---|---|
| O camera | camera | |
| O stethoscope | stethoscope | |
| O blood glucose meter | blood glucose meter | |
| O blood pressure meter | blood pressure meter | |
| O one-canal ECG | one-canal ECG | |
| Patient monitor (PM) | Patient monitor (PM) | |
| O ECG via PM | - ECG via PM | |
| O heart rate via PM | - heart rate via PM | |
| O blood pressure via PM | - blood pressure via PM | |
| O blood oxygen saturation via PM | - blood oxygen saturation via PM | |
| O Temperature via PM | - Temperature via PM | |

**What was difficult when using the TeleDoc devices? What would you like to see?**

***If there is not enough space, use the blank page at the end for further comments.***

# Televisit documentation protocol (3)

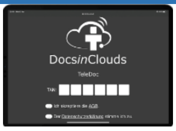


## Rating of the TeleDoc software

How well did you get on with the software?
Provide a school grade from 1 very good to 6 deficient.

What was difficult when using the TeleDoc software? What would you like to see?

## Assessment of the supervision by the GPs

How do you rate the supervision provided by the GPs during the performed televisit?
Please assign a school grade.

| 1 | 2 | 3 | 4 | 5 | 6 |
|---|---|---|---|---|---|
| very good | good | satisfactory | sufficient | poor | deficient |

What would be necessary or desirable to improve the supervision provided by the GPs during this specific televisit?

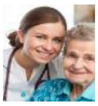

How did the resident feel about the medical care he/ she received during the televisit?
Which school grade did he/she assign for the medical care?

Please enter any comments or remarks from the residents.

# Televisit documentation protocol (4)

## Basic information on the televisit

When was the televisit performed?

| day | | month | |
|---|---|---|---|
| | | | |

The televisit ...

O ... was planned with the GP beforehand.
O ... necessary for an acute issue (non-planned)

Based on what situation or complaint was the televisit performed?

.....................................................................................................................

What would you have done in this specific situation if a televisit had not been available?

O dialed the emergency service via the 112
O dialed the GP out-of-hours service
O waited, until out GP was available

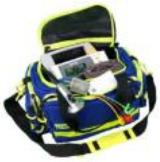

## Use and rating of the integrated point-of-care medical devices

During the televisit ...

O <u>no</u> medical devices
O medical devices

were used.

| Which medical devices integrated into the TeleDoc were used? | How well did you get on with the <u>used</u> devices? Please assign a school grade. | |
|---|---|---|
| O camera | camera | |
| O stethoscope | stethoscope | |
| O blood glucose meter | blood glucose meter | |
| O blood pressure meter | blood pressure meter | |
| O one-canal ECG | one-canal ECG | |
| Patient monitor (PM) | Patient monitor (PM) | |
| O ECG via PM | - ECG via PM | |
| O heart rate via PM | - heart rate via PM | |
| O blood pressure via PM | - blood pressure via PM | |
| O blood oxygen saturation via PM | - blood oxygen saturation via PM | |
| O Temperature via PM | - Temperature via PM | |

What was difficult when using the TeleDoc devices? What would you like to see?

*If there is not enough space, use the blank page at the end for further comments.*

# Televisit documentation protocol (4)

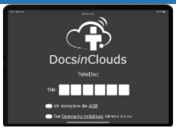


## Rating of the TeleDoc software

How well did you get on with the software?
Provide a school grade from 1 very good to 6 deficient.

What was difficult when using the TeleDoc software? What would you like to see?

## Assessment of the supervision by the GPs

How do you rate the supervision provided by the GPs during the performed televisit?
Please assign a school grade.

| 1 | 2 | 3 | 4 | 5 | 6 |
|---|---|---|---|---|---|
| very good | good | satisfactory | sufficient | poor | deficient |

What would be necessary or desirable to improve the supervision provided by the GPs during this specific televisit?

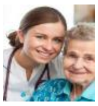

How did the resident feel about the medical care he/ she received during the televisit?
Which school grade did he/she assign for the medical care?

Please enter any comments or remarks from the residents.

# Televisit documentation protocol (5)

## Basic information on the televisit

| | | |
|---|---|---|
| When was the televisit performed? | day | month |
| | | |

The televisit …
- O … was planned with the GP beforehand.
- O … necessary for an acute issue (non-planned)

Based on what situation or complaint was the televisit performed?

……………………………………………………………………………………………………

What would you have done in this specific situation if a televisit had not been available?
- O dialed the emergency service via the 112
- O dialed the GP out-of-hours service
- O waited, until out GP was available

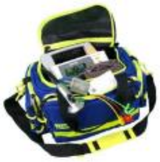

## Use and rating of the integrated point-of-care medical devices

During the televisit …
- O no medical devices were used.
- O medical devices were used.

| Which medical devices integrated into the TeleDoc were used? | How well did you get on with the used devices? Please assign a school grade. | |
|---|---|---|
| O camera | camera | |
| O stethoscope | stethoscope | |
| O blood glucose meter | blood glucose meter | |
| O blood pressure meter | blood pressure meter | |
| O one-canal ECG | one-canal ECG | |
| Patient monitor (PM) | Patient monitor (PM) | |
| O ECG via PM | - ECG via PM | |
| O heart rate via PM | - heart rate via PM | |
| O blood pressure via PM | - blood pressure via PM | |
| O blood oxygen saturation via PM | - blood oxygen saturation via PM | |
| O Temperature via PM | - Temperature via PM | |

What was difficult when using the TeleDoc devices? What would you like to see?

*If there is not enough space, use the blank page at the end for further comments.*

# Televisit documentation protocol (5)

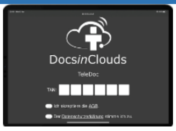


## Rating of the TeleDoc software

How well did you get on with the software?
Provide a school grade from 1 very good to 6 deficient.

What was difficult when using the TeleDoc software? What would you like to see?

## Assessment of the supervision by the GPs

How do you rate the supervision provided by the GPs during the performed televisit?
Please assign a school grade.

| 1 | 2 | 3 | 4 | 5 | 6 |
|---|---|---|---|---|---|
| very good | good | satisfactory | sufficient | poor | deficient |

What would be necessary or desirable to improve the supervision provided by the GPs during this specific televisit?

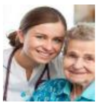

How did the resident feel about the medical care he/ she received during the televisit?
Which school grade did he/she assign for the medical care?

Please enter any comments or remarks from the residents.

# Televisit documentation protocol (6)

## Basic information on the televisit

When was the televisit performed?

| day | | month | |
|---|---|---|---|
| | | | |

The televisit …

O … was planned with the GP beforehand.
O … necessary for an acute issue (non-planned)

Based on what situation or complaint was the televisit performed?

…………………………………………………………………………………………………………………………

What would you have done in this specific situation if a televisit had not been available?

O dialed the emergency service via the 112
O dialed the GP out-of-hours service
O waited, until out GP was available

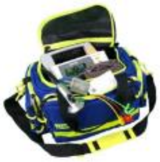

## Use and rating of the integrated point-of-care medical devices

During the televisit …

O <u>no</u> medical devices were used.
O medical devices were used.

| Which medical devices integrated into the TeleDoc were used? | How well did you get on with the <u>used</u> devices? Please assign a school grade. | |
|---|---|---|
| O camera | camera | |
| O stethoscope | stethoscope | |
| O blood glucose meter | blood glucose meter | |
| O blood pressure meter | blood pressure meter | |
| O one-canal ECG | one-canal ECG | |
| Patient monitor (PM) | Patient monitor (PM) | |
| O ECG via PM | - ECG via PM | |
| O heart rate via PM | - heart rate via PM | |
| O blood pressure via PM | - blood pressure via PM | |
| O blood oxygen saturation via PM | - blood oxygen saturation via PM | |
| O Temperature via PM | - Temperature via PM | |

What was difficult when using the TeleDoc devices? What would you like to see?

*If there is not enough space, use the blank page at the end for further comments.*

# Televisit documentation protocol (6)

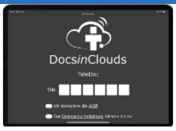


## Rating of the TeleDoc software

How well did you get on with the software?
Provide a school grade from 1 very good to 6 deficient. ☐

What was difficult when using the TeleDoc software? What would you like to see?

## Assessment of the supervision by the GPs

How do you rate the supervision provided by the GPs during the performed televisit?
Please assign a school grade.

| 1 | 2 | 3 | 4 | 5 | 6 |
|---|---|---|---|---|---|
| very good | good | satisfactory | sufficient | poor | deficient |

☐

What would be necessary or desirable to improve the supervision provided by the GPs during this specific televisit?

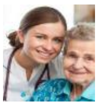

How did the resident feel about the medical care he/ she received during the televisit?
Which school grade did he/she assign for the medical care? ☐

Please enter any comments or remarks from the residents.

# Televisit documentation protocol (7)

## Basic information on the televisit

When was the televisit performed?

| day | | month | |
|---|---|---|---|
| | | | |

The televisit …

- O … was planned with the GP beforehand.
- O … necessary for an acute issue (non-planned)

Based on what situation or complaint was the televisit performed?

…………………………………………………………………………………………………………………………

What would you have done in this specific situation if a televisit had not been available?

- O dialed the emergency service via the 112
- O dialed the GP out-of-hours service
- O waited, until out GP was available

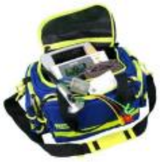

## Use and rating of the integrated point-of-care medical devices

During the televisit …

- O <u>no</u> medical devices
- O medical devices

were used.

| Which medical devices integrated into the TeleDoc were used? | How well did you get on with the <u>used</u> devices? Please assign a school grade. | |
|---|---|---|
| O camera | camera | |
| O stethoscope | stethoscope | |
| O blood glucose meter | blood glucose meter | |
| O blood pressure meter | blood pressure meter | |
| O one-canal ECG | one-canal ECG | |
| Patient monitor (PM) | Patient monitor (PM) | |
| O ECG via PM | - ECG via PM | |
| O heart rate via PM | - heart rate via PM | |
| O blood pressure via PM | - blood pressure via PM | |
| O blood oxygen saturation via PM | - blood oxygen saturation via PM | |
| O Temperature via PM | - Temperature via PM | |

What was difficult when using the TeleDoc devices? What would you like to see?

*If there is not enough space, use the blank page at the end for further comments.*

# Televisit documentation protocol (7)

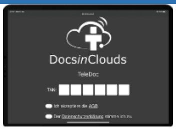


## Rating of the TeleDoc software

How well did you get on with the software?
Provide a school grade from 1 very good to 6 deficient. ☐

What was difficult when using the TeleDoc software? What would you like to see?

## Assessment of the supervision by the GPs

How do you rate the supervision provided by the GPs during the performed televisit?
Please assign a school grade.

| 1 | 2 | 3 | 4 | 5 | 6 |
|---|---|---|---|---|---|
| very good | good | satisfactory | sufficient | poor | deficient |

☐

What would be necessary or desirable to improve the supervision provided by the GPs during this specific televisit?

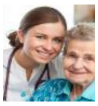

How did the resident feel about the medical care he/ she received during the televisit?
Which school grade did he/she assign for the medical care? ☐

Please enter any comments or remarks from the residents.

# Televisit documentation protocol (8)

## Basic information on the televisit

| When was the televisit performed? | day | | month | |
|---|---|---|---|---|
| | | | | |

The televisit ...

- O ... was planned with the GP beforehand.
- O ... necessary for an acute issue (non-planned)

**Based on what situation or complaint was the televisit performed?**

..........................................................................................................................................

**What would you have done in this specific situation if a televisit had not been available?**

- O dialed the emergency service via the 112
- O dialed the GP out-of-hours service
- O waited, until out GP was available

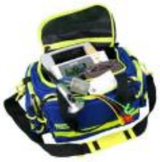

## Use and rating of the integrated point-of-care medical devices

During the televisit ...

- O no medical devices
- O medical devices

were used.

| Which medical devices integrated into the TeleDoc were used? | How well did you get on with the used devices? Please assign a school grade. | |
|---|---|---|
| O camera | camera | |
| O stethoscope | stethoscope | |
| O blood glucose meter | blood glucose meter | |
| O blood pressure meter | blood pressure meter | |
| O one-canal ECG | one-canal ECG | |
| Patient monitor (PM) | Patient monitor (PM) | |
| O ECG via PM | - ECG via PM | |
| O heart rate via PM | - heart rate via PM | |
| O blood pressure via PM | - blood pressure via PM | |
| O blood oxygen saturation via PM | - blood oxygen saturation via PM | |
| O Temperature via PM | - Temperature via PM | |

**What was difficult when using the TeleDoc devices? What would you like to see?**

*If there is not enough space, use the blank page at the end for further comments.*

# Televisit documentation protocol (8)

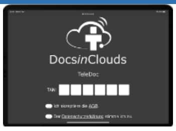


## Rating of the TeleDoc software

How well did you get on with the software?
Provide a school grade from 1 very good to 6 deficient.

What was difficult when using the TeleDoc software? What would you like to see?

## Assessment of the supervision by the GPs

How do you rate the supervision provided by the GPs during the performed televisit?
Please assign a school grade.

| 1 | 2 | 3 | 4 | 5 | 6 |
|---|---|---|---|---|---|
| very good | good | satisfactory | sufficient | poor | deficient |

What would be necessary or desirable to improve the supervision provided by the GPs during this specific televisit?

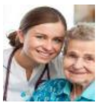

How did the resident feel about the medical care he/ she received during the televisit?
Which school grade did he/she assign for the medical care?

Please enter any comments or remarks from the residents.

# Televisit documentation protocol (9)

## Basic information on the televisit

When was the televisit performed?

| day | | month | |
|---|---|---|---|
| | | | |

The televisit …

O … was planned with the GP beforehand.
O … necessary for an acute issue (non-planned)

Based on what situation or complaint was the televisit performed?

……………………………………………………………………………………………………………………

What would you have done in this specific situation if a televisit had not been available?

O dialed the emergency service via the 112
O dialed the GP out-of-hours service
O waited, until out GP was available

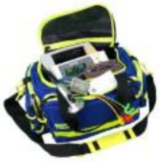

## Use and rating of the integrated point-of-care medical devices

During the televisit …

O <u>no</u> medical devices
O medical devices

were used.

| Which medical devices integrated into the TeleDoc were used? | How well did you get on with the <u>used</u> devices? Please assign a school grade. | |
|---|---|---|
| O camera | camera | |
| O stethoscope | stethoscope | |
| O blood glucose meter | blood glucose meter | |
| O blood pressure meter | blood pressure meter | |
| O one-canal ECG | one-canal ECG | |
| Patient monitor (PM) | Patient monitor (PM) | |
| O ECG via PM | - ECG via PM | |
| O heart rate via PM | - heart rate via PM | |
| O blood pressure via PM | - blood pressure via PM | |
| O blood oxygen saturation via PM | - blood oxygen saturation via PM | |
| O Temperature via PM | - Temperature via PM | |

What was difficult when using the TeleDoc devices? What would you like to see?

*If there is not enough space, use the blank page at the end for further comments.*

# Televisit documentation protocol (9)

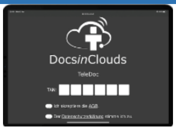


## Rating of the TeleDoc software

How well did you get on with the software?
Provide a school grade from 1 very good to 6 deficient.

What was difficult when using the TeleDoc software? What would you like to see?

## Assessment of the supervision by the GPs

How do you rate the supervision provided by the GPs during the performed televisit?
Please assign a school grade.

| 1 | 2 | 3 | 4 | 5 | 6 |
|---|---|---|---|---|---|
| very good | good | satisfactory | sufficient | poor | deficient |

What would be necessary or desirable to improve the supervision provided by the GPs during this specific televisit?

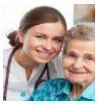

How did the resident feel about the medical care he/ she received during the televisit?
Which school grade did he/she assign for the medical care?

Please enter any comments or remarks from the residents.

# Televisit documentation protocol (10)

## Basic information on the televisit

| When was the televisit performed? | day | | month | |
|---|---|---|---|---|
| | | | | |

The televisit …

- O … was planned with the GP beforehand.
- O … necessary for an acute issue (non-planned)

Based on what situation or complaint was the televisit performed?

………………………………………………………………………………………………………………………

What would you have done in this specific situation if a televisit had not been available?

- O dialed the emergency service via the 112
- O dialed the GP out-of-hours service
- O waited, until out GP was available

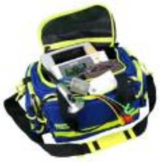

## Use and rating of the integrated point-of-care medical devices

During the televisit …

- O <u>no</u> medical devices
- O medical devices

were used.

| Which medical devices integrated into the TeleDoc were used? | How well did you get on with the <u>used</u> devices? Please assign a school grade. | |
|---|---|---|
| O camera | camera | |
| O stethoscope | stethoscope | |
| O blood glucose meter | blood glucose meter | |
| O blood pressure meter | blood pressure meter | |
| O one-canal ECG | one-canal ECG | |
| Patient monitor (PM) | Patient monitor (PM) | |
| O ECG via PM | - ECG via PM | |
| O heart rate via PM | - heart rate via PM | |
| O blood pressure via PM | - blood pressure via PM | |
| O blood oxygen saturation via PM | - blood oxygen saturation via PM | |
| O Temperature via PM | - Temperature via PM | |

What was difficult when using the TeleDoc devices? What would you like to see?

*If there is not enough space, use the blank page at the end for further comments.*

# Televisit documentation protocol (10)

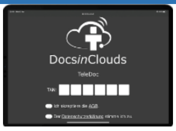


## Rating of the TeleDoc software

How well did you get on with the software?
Provide a school grade from 1 very good to 6 deficient.

What was difficult when using the TeleDoc software? What would you like to see?

## Assessment of the supervision by the GPs

How do you rate the supervision provided by the GPs during the performed televisit?
Please assign a school grade.

| 1 | 2 | 3 | 4 | 5 | 6 |
|---|---|---|---|---|---|
| very good | good | satisfactory | sufficient | poor | deficient |

What would be necessary or desirable to improve the supervision provided by the GPs during this specific televisit?

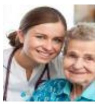

How did the resident feel about the medical care he/ she received during the televisit?
Which school grade did he/she assign for the medical care?

Please enter any comments or remarks from the residents.

# Space for additionnal comments

**For comments, please indicate the date the televisit was performed!**

# Question to the residents

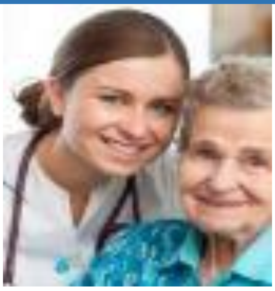

Please ask the resident as soon as possible after the televisit:

1) How did you feel about the medical care during the televisit?

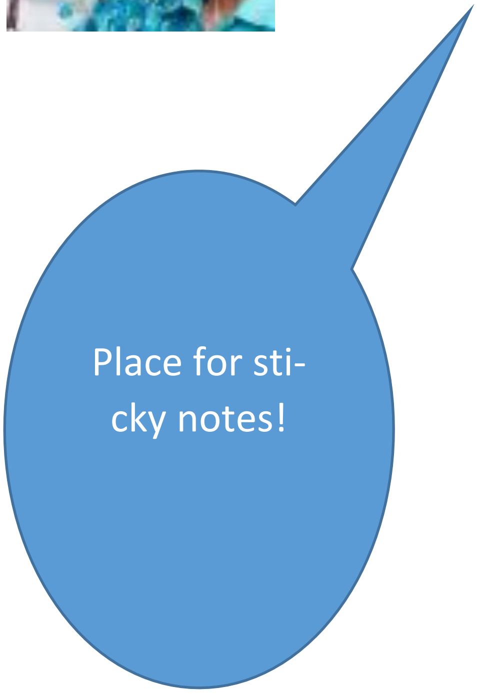


2) As a patient, how would you rate the medical care you received during the televisit ?

Say what each grade means and, if necessery, show the shool grades with the smilies.

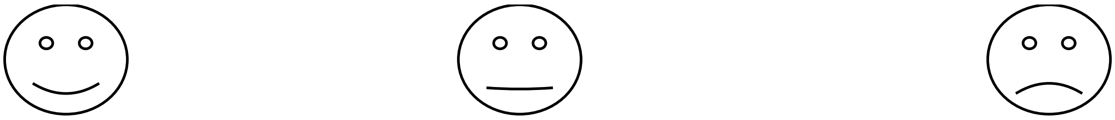

| 1 | 2 | 3 | 4 | 5 | 6 |
|---|---|---|---|---|---|
| very good | good | satisfactory | sufficient | poor | deficient |

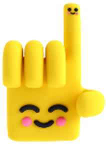

**Enter the comments and the school grade at the end of each protocol for a televisit!**

# AIDA

## Arbeitsentwicklung In Der Altenpflege

**Information concerning this project at www.projekt-aida.org**

| | |
|---|---|
| Logo of a project partner | Logo of a project partner |
| Logo of a project partner | Logo of a project partner |
| Logo of a project partner | Logo of a project partner |
| Logo of a project partner | Logo of a project partner |

EUROPÄISCHE UNION
Investition in unsere Zukunft
Europäischer Fonds
für regionale Entwicklung

Die Landesregierung
Nordrhein-Westfalen

Future of elderly care

Logo eines Projektpart-
ners

# Projekttagebuch

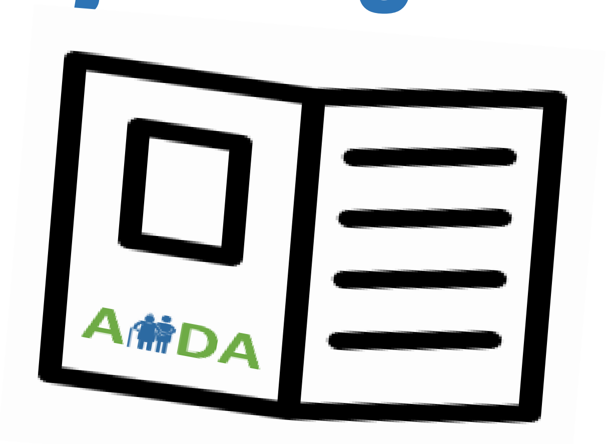


## Ausgabe 1

***Hinweis für das Pflegeheim:***

*Bitte tragen Sie den Personencode vor der Übergabe an die Pflegekraft ein!*

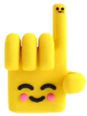

| **Code** | 1 | 1 | | |
|---|---|---|---|---|

***Hinweis:***
*Bitte schneiden Sie die untere Ecke mit Ihrem Namen vor der Rückgabe ab.*

**Dieses Pflegetagebuch gehört:**

## Projekt & Tagebuch – Auf einen Blick!

**Ziel:**
Mit dem Projekt AIDA soll ein praxistaugliches Konzept für Televisiten in stationären Altenpflegeeinrichtungen entwickelt werden.

**Weg:**
Dafür werden im Projekt bereits in der Entwicklungsphase Nutzer in den Pflegeheimen und Arztpraxis eingebunden.

**Methode:**
Für eine systematische Erfassung der Hinweise und Anregungen aus der Praxis hat MA&T ein Projekttagebuch entwickelt.

**Aufbau:**
Das Projekttagebuch umfasst verschiedene – farbige – Teile.

**Ihre Ansprechpartnerin für Fragen zum Projekttagebuch:**

**XXXXXXXXXX**
Telefon: xxxxxxxxxxxxxxxxx
mailvonkontaktperson@xxxxxx

**Grunddaten:**
**Angaben zu Geschlecht, Alter, Berufserfahrungen etc. werden nur einmal erhoben.**

**Informationen: Dieser Teil ist nur zu Beginn der Erprobung auszufüllen und bei neuen Schulungen oder Anleitungen zu ergänzen.**

**Einsatzprotokolle für jede Televisite:**
**Das kurze Protokoll ist möglich zeitnah nach jedem Einsatz auszufüllen.**

**Wir wollen – mit Ihrer Unterstützung – auch erfassen, wie die Bewohner/innen die ärztliche Betreuung bei der Televisiten fanden! Bitte fragen Sie danach und tragen Sie die Antwort am Ende des Einsatzprotokolls ein. Auf der letzten Heftseite finden Sie die Frage an die Bewohner/innen, die Schulnoten-Bewertung mit den Smilies sowie Platz für Klebezettel-Notizen.**

## Hilfen zur Nutzung:

**Visualisierung durch Piktogramme und eindeutige Symbole vereinfachen das Ausfüllen:**

**Dieses Symbol verweist auf Hinweise.**

O **Kreise werden verwendet, um Zutreffendes anzukreuzen.**

**Rechtecke werden verwendet für die Eingabe von Ziffern (z.B. Zeitangaben, Bewertungen)**

**Bewertungen erfolgen auf Grundlage der vertrauten Schulnoten.**

| 1 | 2 | 3 | 4 | 5 | 6 |
|---|---|---|---|---|---|
| sehr gut | gut | befriedigend | ausreichend | mangelhaft | ungenügend |

**Benutzen Sie zum Ausfüllen möglichst einen Filzschreiber oder einen Kugelschreiber, bitte keinen Bleistift.**

# Grunddaten

**Bitte geben Sie uns Auskunft zu folgenden Punkten.**

**Ich bin …** O **weiblich** O **männlich** **Ich bin …** ☐ **Jahre alt.**

**Ich verfüge über** ☐ **Jahre Berufserfahrung** (ohne Ausbildungszeiten) **in der Pflege bzw. im medizinischen Bereich.**

**Ich bin …**

O Pflegehilfskraft

O Pflegefachkraft

O etwas anderes und zwar:

O Wohnbereichsleiter/in

O Pflegedienstleiter/in

**Für den Einsatz der Televisite in unserem Pflegeheim übernehme ich besondere Aufgaben** (z.B. Mitarbeitereinweisung, Durchführung der Updates).

O **Ja, folgende Aufgaben: ……………………………………………………..……………**

**Wie schätzen Sie Ihre Kompetenzen im Umgang mit medizintechnischen Geräten ein, die bisher im Pflegeheim eingesetzt werden. Bitte vergeben Sie eine Schulnote.**

☺ 😐 ☹

| 1 | 2 | 3 | 4 | 5 | 6 |
|---|---|---|---|---|---|
| sehr gut | gut | befriedigend | ausreichend | mangelhaft | ungenügend |

☐

**Wie schätzen Sie Ihre Kompetenzen im Umgang mit Computerprogrammen (z.B. zur Pflegedokumentation) ein? Bitte vergeben Sie eine Note.** ☐

**Welche Erwartungen verbinden Sie mit dem Einsatz von Televisiten für die ärztliche Versorgung der Bewohner? (Kreuzen Sie alle Aussagen an, denen Sie zustimmen)**

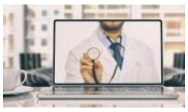

O Die ärztliche Versorgung kann damit deutlich verbessert werden.

O Positive und negative Aspekte werden in etwa gleich sein.

O Televisiten werden bei unseren Bewohnern schwierig sein.

O Televisiten sollten nur im Ausnahmefall genutzt werden.

**Welche Erwartungen verbinden Sie mit dem Einsatz von Televisiten für Ihre Arbeit? Die Klärung gesundheitlicher Belange von Bewohnern wird damit voraussichtlich …**

O zeitlich weniger aufwendig

O nur anders, aber zeitlich insgesamt ebenso aufwendig

O aufwendiger

**werden?** Bitte die zutreffende Antwort ankreuzen!

# Informationen – zur TeleDoc-Software

**Kreuzen Sie bitte an, zu welchen Punkten Sie Informationen erhalten haben. Beantworten Sie für diese Punkte bitte einige weitere Fragen.**

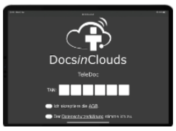


**Ich habe Informationen zur TeleDoc-Software erhalten.**

O **Ja**
O **Nein**

**Ich habe diese Informationen erhalten über ...** (mehrfaches Ankreuzen möglich!)

O **eine Einweisung Vorort am PC**
- O durch Mitarbeiter des Projekts
- O durch Mitarbeiter des Pflegeheims

O **eine digitale Einweisung am PC**
- O durch Mitarbeiter des Projekts
- O durch Mitarbeiter des Pflegeheims

O **digitale Schulungen / Medien**
- O Videos
- O Online Präsentationen

O **schriftliche Informationen**
- O ein Handbuch
- O andere Unterlagen, und zwar

.....................................................

**Wie gut fühlen Sie sich zur TeleDoc-Software aktuell am .... informiert?**
**Vergeben Sie eine Schulnote.**

| Tag | | Monat | |
|---|---|---|---|
| | | | |

| 1 | 2 | 3 | 4 | 5 | 6 |
|---|---|---|---|---|---|
| sehr gut | gut | befriedigend | ausreichend | mangelhaft | ungenügend |

**Was war bei den Informationen zur TeleDoc-Software schwierig zu verstehen oder kam zu kurz? Was wünschen Sie sich?**

# Informationen – zu TeleDoc-Medizingeräten

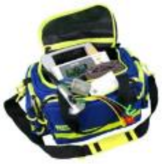

**Ich habe Informationen zu TeleDoc-Medizingeräten erhalten.** O **Ja** O **Nein**

**Ich habe diese Informationen erhalten über …** (mehrfaches Ankreuzen möglich!)

| | |
|---|---|
| O **eine Einweisung Vorort** | O durch Mitarbeiter des Projekts<br>O durch Mitarbeiter des Pflegeheims |
| O **digitale Schulungen / Medien** | O Videos<br>O Online Präsentationen |
| O **schriftliche Informationen** | O ein Handbuch<br>O andere Unterlagen, und zwar<br>............................................................... |

| **Welche TeleDoc-Geräte sind vorhanden?** | **Zu welchen Geräten haben Sie Informationen erhalten?** | **Bewerten Sie jeweils die Informationen mit einer Schulnote.** | | **Visualisierte Kurzanleitung wäre gut für:** |
|---|---|---|---|---|
| O Kamera | O Kamera | Kamera | | O |
| O Stethoskop | O Stethoskop | Stethoskop | | O |
| O Blutzuckergerät | O Blutzuckergerät | Blutzucker-Gerät | | O |
| O Blutdruckgerät | O Blutdruckgerät | Blutdruckgerät | | O |
| O Pocket WiWe EKG | O Pocket WiWe EKG | Pocket WiWe EKG | | O |
| O Atemwegscanner | O Atemwegscanner | Atemwegscanner | | O |
| O Ultraschallgerät | O Ultraschallgerät | Ultraschallgerät | | O |
| O Patientenmonitor | O Patientenmonitor | Patientenmonitor | | O und zwar für<br>............................................................... |

(mit EKG-, Temperatur-, Puls-, Blutdruck-Sauerstoffsättigungs-, Messgerät)

**Was war bei den Informationen zu TeleDoc-Geräten schwierig zu verstehen oder kam zu kurz? Was wünschen Sie sich?**

***Glückwunsch! Die Befragungsteile zu Erprobungsbeginn sind geschafft!*** 😊
***Es folgen 10 Einsatzprotokolle! Melden Sie sich, wenn Sie mehr brauchen!***

# Einsatzprotokoll zur Televisite (1)

## Grunddaten zur Televisite

| Wann fand die Televisite statt? | Tag | | Monat | |
|---|---|---|---|---|
| | | | | |

Es handelte sich um …

- O … eine mit dem Arzt vorher geplante Televisite.
- O … eine aktuell erforderliche Televisite

**Aufgrund welcher Situation bzw. Beschwerden wurde die Televisite durchgeführt?**

……………………………………………………………………………………………………

**Was hätten Sie in dem konkreten Fall ohne Telemedizin unternommen?**

- O den Notruf 112 angerufen
- O den ärztlichen Bereitschaftsdienst angerufen
- O abgewartet, bis unser Arzt kommen kann

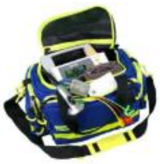

## Einsatz und Bewertung der TeleDoc-Medizingeräte

Bei der Televisite kamen …

- O <u>keine</u> TeleDoc-Medizingeräte
- O TeleDoc-Doc-Medizingeräte

zum Einsatz.

| Welche TeleDoc-Doc-Medizingeräte wurden eingesetzt! | Wie gut sind Sie mit den <u>eingesetzten</u> Geräten zurechtgekommen? Vergeben Sie eine Note. | |
|---|---|---|
| O Kamera | Kamera | |
| O Stethoskop | Stethoskop | |
| O Blutzuckergerät | Blutzuckergerät | |
| O Blutdruckgerät | Blutdruckgerät | |
| O Pocket WiWe EKG | Pocket WiWe EKG | |
| Patientenmonitor (PM) | Patientenmonitor (PM) | |
| O EKG über PM | - EKG über PM | |
| O Puls über PM | - Puls über PM | |
| O Blutdruck über PM | - Blutdruck über PM | |
| O Sauerstoffsättigung über PM | - Sauerstoffsättigung über PM | |
| O Temperatur über PM | - Temperatur über PM | |

**Was war beim Einsatz von TeleDoc-Geräten schwierig? Was wünschen Sie sich?**

***Falls der Platz nicht reicht, nutzen Sie für weitere Kommentare die leere Seite hinten.***

# Einsatzprotokoll zur Televisite (1)

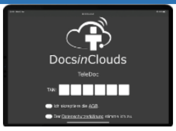


## Bewertung der TeleDoc-Software

**Wie gut sind Sie mit der TeleDoc-Software zu Recht gekommen?**
**Vergeben Sie eine Schulnote von 1 sehr gut bis 6 unzureichend.** ☐

**Was war bei der Nutzung der TeleDoc-Software schwierig? Was wünschen Sie sich?**

## Einschätzung der ärztlichen Betreuung bei der Televisite

**Wie schätzen Sie die ärztliche Betreuung bei der durchgeführten Televisite ein?**
**Vergeben Sie eine Schulnote.**

| 1 | 2 | 3 | 4 | 5 | 6 |
|---|---|---|---|---|---|
| sehr gut | gut | befriedigend | ausreichend | mangelhaft | ungenügend |

☐

**Was wäre für den konkreten Fall für ein bessere ärtzliche Versorgung per Televisite erforderlich bzw. wünschenswert?**

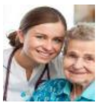

**Wie fand der bzw. die Bewohner/in die ärztliche Betreuung bei der Televiste?**
**Welche Schulnote wurden für die ärztliche Betreuung vergeben?** ☐

**Tragen Sie bitte Anmerkungen oder Hinweise des Bewohners der Bewohnerin ein.**

# Einsatzprotokoll zur Televisite (2)

## Grunddaten zur Televisite

| Wann fand die Televisite statt? | Tag | | Monat | |
|---|---|---|---|---|
| | | | | |

Es handelte sich um …

O … eine mit dem Arzt vorher geplante Televisite.
O … eine aktuell erforderliche Televisite

**Aufgrund welcher Situation bzw. Beschwerden wurde die Televisite durchgeführt?**

………………………………………………………………………………………………

**Was hätten Sie in dem konkreten Fall ohne Telemedizin unternommen?**

O den Notruf 112 angerufen
O den ärztlichen Bereitschaftsdienst angerufen
O abgewartet, bis unser Arzt kommen kann

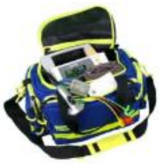

## Einsatz und Bewertung der TeleDoc-Medizingeräte

Bei der Televisite kamen …

O keine TeleDoc-Medizingeräte
O TeleDoc-Doc-Medizingeräte

zum Einsatz.

| Welche TeleDoc-Doc-Medizingeräte wurden eingesetzt! | Wie gut sind Sie mit den eingesetzten Geräten zurechtgekommen? Vergeben Sie eine Note. | |
|---|---|---|
| O Kamera | Kamera | |
| O Stethoskop | Stethoskop | |
| O Blutzuckergerät | Blutzuckergerät | |
| O Blutdruckgerät | Blutdruckgerät | |
| O Pocket WiWe EKG | Pocket WiWe EKG | |
| Patientenmonitor (PM) | Patientenmonitor (PM) | |
| O EKG über PM | - EKG über PM | |
| O Puls über PM | - Puls über PM | |
| O Blutdruck über PM | - Blutdruck über PM | |
| O Sauerstoffsättigung über PM | - Sauerstoffsättigung über PM | |
| O Temperatur über PM | - Temperatur über PM | |

**Was war beim Einsatz von TeleDoc-Geräten schwierig? Was wünschen Sie sich?**

# Einsatzprotokoll zur Televisite (2)

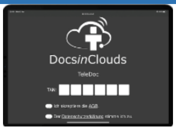


## Bewertung der TeleDoc-Software

**Wie gut sind Sie mit der TeleDoc-Software zu Recht gekommen?**
**Vergeben Sie eine Schulnote von 1 sehr gut bis 6 unzureichend.** ☐

**Was war bei der Nutzung der TeleDoc-Software schwierig? Was wünschen Sie sich?**

## Einschätzung der ärztlichen Betreuung bei der Televisite

**Wie schätzen Sie die ärztliche Betreuung bei der durchgeführten Televisite ein?**
**Vergeben Sie eine Schulnote.**

| 1 | 2 | 3 | 4 | 5 | 6 |
|---|---|---|---|---|---|
| sehr gut | gut | befriedigend | ausreichend | mangelhaft | ungenügend |

☐

**Was wäre für den konkreten Fall für ein bessere ärtzliche Versorgung per Televisite erforderlich bzw. wünschenswert?**

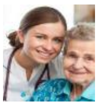

**Wie fand der bzw. die Bewohner/in die ärztliche Betreuung bei der Televiste?**
**Welche Schulnote wurden für die ärztliche Betreuung vergeben?** ☐

**Tragen Sie bitte Anmerkungen oder Hinweise des Bewohners der Bewohnerin ein.**

# Einsatzprotokoll zur Televisite (3)

## Grunddaten zur Televisite

| Wann fand die Televisite statt? | Tag | | Monat | |
|---|---|---|---|---|
| | | | | |

**Es handelte sich um …**

O … eine mit dem Arzt vorher geplante Televisite.
O … eine aktuell erforderliche Televisite

**Aufgrund welcher Situation bzw. Beschwerden wurde die Televisite durchgeführt?**

……………………………………………………………………………………………………

**Was hätten Sie in dem konkreten Fall ohne Telemedizin unternommen?**

O den Notruf 112 angerufen
O den ärztlichen Bereitschaftsdienst angerufen
O abgewartet, bis unser Arzt kommen kann

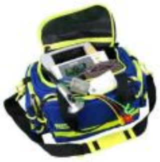

## Einsatz und Bewertung der TeleDoc-Medizingeräte

**Bei der Televisite kamen …**

O <u>keine</u> TeleDoc-Medizingeräte
O TeleDoc-Doc-Medizingeräte

**zum Einsatz.**

| **Welche TeleDoc-Doc-Medizingeräte wurden eingesetzt!** | **Wie gut sind Sie mit den <u>eingesetzten</u> Geräten zurechtgekommen? Vergeben Sie eine Note.** | |
|---|---|---|
| O Kamera | Kamera | |
| O Stethoskop | Stethoskop | |
| O Blutzuckergerät | Blutzuckergerät | |
| O Blutdruckgerät | Blutdruckgerät | |
| O Pocket WiWe EKG | Pocket WiWe EKG | |
| Patientenmonitor (PM) | Patientenmonitor (PM) | |
| O EKG über PM | - EKG über PM | |
| O Puls über PM | - Puls über PM | |
| O Blutdruck über PM | - Blutdruck über PM | |
| O Sauerstoffsättigung über PM | - Sauerstoffsättigung über PM | |
| O Temperatur über PM | - Temperatur über PM | |

**Was war beim Einsatz von TeleDoc-Geräten schwierig? Was wünschen Sie sich?**

# Einsatzprotokoll zur Televisite (3)

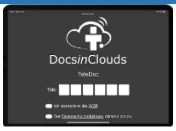


## Bewertung der TeleDoc-Software

**Wie gut sind Sie mit der TeleDoc-Software zu Recht gekommen?**
**Vergeben Sie eine Schulnote von 1 sehr gut bis 6 unzureichend.** ☐

**Was war bei der Nutzung der TeleDoc-Software schwierig? Was wünschen Sie sich?**

## Einschätzung der ärztlichen Betreuung bei der Televisite

**Wie schätzen Sie die ärztliche Betreuung bei der durchgeführten Televisite ein?**
**Vergeben Sie eine Schulnote.**

| 1 | 2 | 3 | 4 | 5 | 6 |
|---|---|---|---|---|---|
| sehr gut | gut | befriedigend | ausreichend | mangelhaft | ungenügend |

☐

**Was wäre für den konkreten Fall für ein bessere ärtzliche Versorgung per Televisite erforderlich bzw. wünschenswert?**

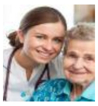

**Wie fand der bzw. die Bewohner/in die ärztliche Betreuung bei der Televiste?**
**Welche Schulnote wurden für die ärztliche Betreuung vergeben?** ☐

**Tragen Sie bitte Anmerkungen oder Hinweise des Bewohners der Bewohnerin ein.**

# Einsatzprotokoll zur Televisite (4)

## Grunddaten zur Televisite

| Wann fand die Televisite statt? | Tag | | Monat | |
|---|---|---|---|---|
| | | | | |

Es handelte sich um …

- O … eine mit dem Arzt vorher geplante Televisite.
- O … eine aktuell erforderliche Televisite

**Aufgrund welcher Situation bzw. Beschwerden wurde die Televisite durchgeführt?**

……………………………………………………………………………………………

Was hätten Sie in dem konkreten Fall ohne Telemedizin unternommen?

- O den Notruf 112 angerufen
- O den ärztlichen Bereitschaftsdienst angerufen
- O abgewartet, bis unser Arzt kommen kann

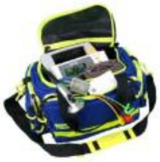

## Einsatz und Bewertung der TeleDoc-Medizingeräte

Bei der Televisite kamen …

- O <u>keine</u> TeleDoc-Medizingeräte
- O TeleDoc-Doc-Medizingeräte

zum Einsatz.

| Welche TeleDoc-Doc-Medizingeräte wurden eingesetzt! | Wie gut sind Sie mit den <u>eingesetzten</u> Geräten zurechtgekommen? Vergeben Sie eine Note. | |
|---|---|---|
| O Kamera | Kamera | |
| O Stethoskop | Stethoskop | |
| O Blutzuckergerät | Blutzuckergerät | |
| O Blutdruckgerät | Blutdruckgerät | |
| O Pocket WiWe EKG | Pocket WiWe EKG | |
| Patientenmonitor (PM) | Patientenmonitor (PM) | |
| O EKG über PM | - EKG über PM | |
| O Puls über PM | - Puls über PM | |
| O Blutdruck über PM | - Blutdruck über PM | |
| O Sauerstoffsättigung über PM | - Sauerstoffsättigung über PM | |
| O Temperatur über PM | - Temperatur über PM | |

**Was war beim Einsatz von TeleDoc-Geräten schwierig? Was wünschen Sie sich?**

# Einsatzprotokoll zur Televisite (4)

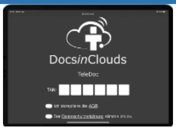


## Bewertung der TeleDoc-Software

**Wie gut sind Sie mit der TeleDoc-Software zu Recht gekommen?**
**Vergeben Sie eine Schulnote von 1 sehr gut bis 6 unzureichend.** ☐

**Was war bei der Nutzung der TeleDoc-Software schwierig? Was wünschen Sie sich?**

## Einschätzung der ärztlichen Betreuung bei der Televisite

**Wie schätzen Sie die ärztliche Betreuung bei der durchgeführten Televisite ein?**
**Vergeben Sie eine Schulnote.**

| 1 | 2 | 3 | 4 | 5 | 6 |
|---|---|---|---|---|---|
| sehr gut | gut | befriedigend | ausreichend | mangelhaft | ungenügend |

☐

**Was wäre für den konkreten Fall für ein bessere ärtzliche Versorgung per Televisite erforderlich bzw. wünschenswert?**

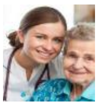

**Wie fand der bzw. die Bewohner/in die ärztliche Betreuung bei der Televiste?**
**Welche Schulnote wurden für die ärztliche Betreuung vergeben?** ☐

**Tragen Sie bitte Anmerkungen oder Hinweise des Bewohners der Bewohnerin ein.**

# Einsatzprotokoll zur Televisite (5)

## Grunddaten zur Televisite

| Wann fand die Televisite statt? | Tag | | Monat | |
|---|---|---|---|---|
| | | | | |

Es handelte sich um …
- O … eine mit dem Arzt vorher geplante Televisite.
- O … eine aktuell erforderliche Televisite

**Aufgrund welcher Situation bzw. Beschwerden wurde die Televisite durchgeführt?**

……………………………………………………………………………………………………

**Was hätten Sie in dem konkreten Fall ohne Telemedizin unternommen?**
- O den Notruf 112 angerufen
- O den ärztlichen Bereitschaftsdienst angerufen
- O abgewartet, bis unser Arzt kommen kann

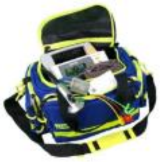

## Einsatz und Bewertung der TeleDoc-Medizingeräte

Bei der Televisite kamen …
- O <u>keine</u> TeleDoc-Medizingeräte
- O TeleDoc-Doc-Medizingeräte

zum Einsatz.

| Welche TeleDoc-Doc-Medizingeräte wurden eingesetzt! | Wie gut sind Sie mit den <u>eingesetzten</u> Geräten zurechtgekommen? Vergeben Sie eine Note. | |
|---|---|---|
| O Kamera | Kamera | |
| O Stethoskop | Stethoskop | |
| O Blutzuckergerät | Blutzuckergerät | |
| O Blutdruckgerät | Blutdruckgerät | |
| O Pocket WiWe EKG | Pocket WiWe EKG | |
| Patientenmonitor (PM) | Patientenmonitor (PM) | |
| O EKG über PM | - EKG über PM | |
| O Puls über PM | - Puls über PM | |
| O Blutdruck über PM | - Blutdruck über PM | |
| O Sauerstoffsättigung über PM | - Sauerstoffsättigung über PM | |
| O Temperatur über PM | - Temperatur über PM | |

**Was war beim Einsatz von TeleDoc-Geräten schwierig? Was wünschen Sie sich?**

# Einsatzprotokoll zur Televisite (5)

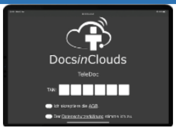


## Bewertung der TeleDoc-Software

**Wie gut sind Sie mit der TeleDoc-Software zu Recht gekommen?**
**Vergeben Sie eine Schulnote von 1 sehr gut bis 6 unzureichend.** ☐

**Was war bei der Nutzung der TeleDoc-Software schwierig? Was wünschen Sie sich?**

## Einschätzung der ärztlichen Betreuung bei der Televisite

**Wie schätzen Sie die ärztliche Betreuung bei der durchgeführten Televisite ein?**
**Vergeben Sie eine Schulnote.**

| 1 | 2 | 3 | 4 | 5 | 6 |
|---|---|---|---|---|---|
| sehr gut | gut | befriedigend | ausreichend | mangelhaft | ungenügend |

☐

**Was wäre für den konkreten Fall für ein bessere ärtzliche Versorgung per Televisite erforderlich bzw. wünschenswert?**

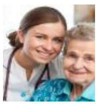

**Wie fand der bzw. die Bewohner/in die ärztliche Betreuung bei der Televiste?**
**Welche Schulnote wurden für die ärztliche Betreuung vergeben?** ☐

**Tragen Sie bitte Anmerkungen oder Hinweise des Bewohners der Bewohnerin ein.**

# Einsatzprotokoll zur Televisite (6)

## Grunddaten zur Televisite

| Wann fand die Televisite statt? | Tag | | Monat | |
|---|---|---|---|---|
| | | | | |

Es handelte sich um …

- O … eine mit dem Arzt vorher geplante Televisite.
- O … eine aktuell erforderliche Televisite

**Aufgrund welcher Situation bzw. Beschwerden wurde die Televisite durchgeführt?**

…………………………………………………………………………………………………………………………

**Was hätten Sie in dem konkreten Fall ohne Telemedizin unternommen?**

- O den Notruf 112 angerufen
- O den ärztlichen Bereitschaftsdienst angerufen
- O abgewartet, bis unser Arzt kommen kann

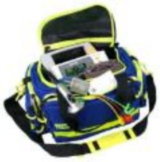

## Einsatz und Bewertung der TeleDoc-Medizingeräte

Bei der Televisite kamen …

- O <u>keine</u> TeleDoc-Medizingeräte
- O TeleDoc-Doc-Medizingeräte

zum Einsatz.

| Welche TeleDoc-Doc-Medizingeräte wurden eingesetzt! | Wie gut sind Sie mit den <u>eingesetzten</u> Geräten zurechtgekommen? Vergeben Sie eine Note. | |
|---|---|---|
| O Kamera | Kamera | |
| O Stethoskop | Stethoskop | |
| O Blutzuckergerät | Blutzuckergerät | |
| O Blutdruckgerät | Blutdruckgerät | |
| O Pocket WiWe EKG | Pocket WiWe EKG | |
| Patientenmonitor (PM) | Patientenmonitor (PM) | |
| O EKG über PM | - EKG über PM | |
| O Puls über PM | - Puls über PM | |
| O Blutdruck über PM | - Blutdruck über PM | |
| O Sauerstoffsättigung über PM | - Sauerstoffsättigung über PM | |
| O Temperatur über PM | - Temperatur über PM | |

**Was war beim Einsatz von TeleDoc-Geräten schwierig? Was wünschen Sie sich?**

# Einsatzprotokoll zur Televisite (6)

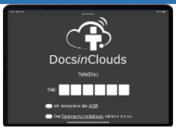


## Bewertung der TeleDoc-Software

**Wie gut sind Sie mit der TeleDoc-Software zu Recht gekommen?**
**Vergeben Sie eine Schulnote von 1 sehr gut bis 6 unzureichend.**

**Was war bei der Nutzung der TeleDoc-Software schwierig? Was wünschen Sie sich?**

## Einschätzung der ärztlichen Betreuung bei der Televisite

**Wie schätzen Sie die ärztliche Betreuung bei der durchgeführten Televisite ein?**
**Vergeben Sie eine Schulnote.**

| 1 | 2 | 3 | 4 | 5 | 6 |
|---|---|---|---|---|---|
| sehr gut | gut | befriedigend | ausreichend | mangelhaft | ungenügend |

**Was wäre für den konkreten Fall für ein bessere ärtzliche Versorgung per Televisite erforderlich bzw. wünschenswert?**

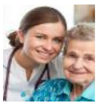

**Wie fand der bzw. die Bewohner/in die ärztliche Betreuung bei der Televiste?**
**Welche Schulnote wurden für die ärztliche Betreuung vergeben?**

**Tragen Sie bitte Anmerkungen oder Hinweise des Bewohners der Bewohnerin ein.**

# Einsatzprotokoll zur Televisite (7)

## Grunddaten zur Televisite

**Wann fand die Televisite statt?**

| Tag | | Monat | |
|---|---|---|---|
| | | | |

**Es handelte sich um …**

O … eine mit dem Arzt vorher geplante Televisite.
O … eine aktuell erforderliche Televisite

**Aufgrund welcher Situation bzw. Beschwerden wurde die Televisite durchgeführt?**

……………………………………………………………………………………………………

**Was hätten Sie in dem konkreten Fall ohne Telemedizin unternommen?**

O den Notruf 112 angerufen
O den ärztlichen Bereitschaftsdienst angerufen
O abgewartet, bis unser Arzt kommen kann

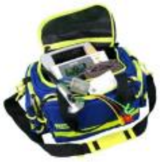

## Einsatz und Bewertung der TeleDoc-Medizingeräte

**Bei der Televisite kamen …**

O keine TeleDoc-Medizingeräte zum Einsatz.
O TeleDoc-Doc-Medizingeräte zum Einsatz.

| Welche TeleDoc-Doc-Medizingeräte wurden eingesetzt! | Wie gut sind Sie mit den eingesetzten Geräten zurechtgekommen? Vergeben Sie eine Note. | |
|---|---|---|
| O Kamera | Kamera | |
| O Stethoskop | Stethoskop | |
| O Blutzuckergerät | Blutzuckergerät | |
| O Blutdruckgerät | Blutdruckgerät | |
| O Pocket WiWe EKG | Pocket WiWe EKG | |
| Patientenmonitor (PM) | Patientenmonitor (PM) | |
| O EKG über PM | - EKG über PM | |
| O Puls über PM | - Puls über PM | |
| O Blutdruck über PM | - Blutdruck über PM | |
| O Sauerstoffsättigung über PM | - Sauerstoffsättigung über PM | |
| O Temperatur über PM | - Temperatur über PM | |

**Was war beim Einsatz von TeleDoc-Geräten schwierig? Was wünschen Sie sich?**

# Einsatzprotokoll zur Televisite (7)

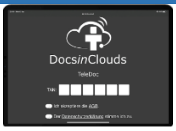


## Bewertung der TeleDoc-Software

**Wie gut sind Sie mit der TeleDoc-Software zu Recht gekommen?**
**Vergeben Sie eine Schulnote von 1 sehr gut bis 6 unzureichend.** ☐

**Was war bei der Nutzung der TeleDoc-Software schwierig? Was wünschen Sie sich?**

## Einschätzung der ärztlichen Betreuung bei der Televisite

**Wie schätzen Sie die ärztliche Betreuung bei der durchgeführten Televisite ein?**
**Vergeben Sie eine Schulnote.**

| 1 | 2 | 3 | 4 | 5 | 6 |
|---|---|---|---|---|---|
| sehr gut | gut | befriedigend | ausreichend | mangelhaft | ungenügend |

☐

**Was wäre für den konkreten Fall für ein bessere ärtzliche Versorgung per Televisite erforderlich bzw. wünschenswert?**

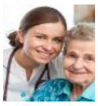

**Wie fand der bzw. die Bewohner/in die ärztliche Betreuung bei der Televiste?**
**Welche Schulnote wurden für die ärztliche Betreuung vergeben?** ☐

**Tragen Sie bitte Anmerkungen oder Hinweise des Bewohners der Bewohnerin ein.**

# Einsatzprotokoll zur Televisite (8)

## Grunddaten zur Televisite

**Wann fand die Televisite statt?**

| Tag | | Monat | |
|---|---|---|---|
| | | | |

**Es handelte sich um …**

O **… eine mit dem Arzt vorher geplante Televisite.**
O **… eine aktuell erforderliche Televisite**

**Aufgrund welcher Situation bzw. Beschwerden wurde die Televisite durchgeführt?**

……………………………………………………………………………………………………

**Was hätten Sie in dem konkreten Fall ohne Telemedizin unternommen?**

O **den Notruf 112 angerufen**
O **den ärztlichen Bereitschaftsdienst angerufen**
O **abgewartet, bis unser Arzt kommen kann**

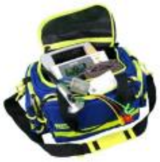

## Einsatz und Bewertung der TeleDoc-Medizingeräte

**Bei der Televisite kamen …**

O **<u>keine</u> TeleDoc-Medizingeräte**
O **TeleDoc-Doc-Medizingeräte**

**zum Einsatz.**

| **Welche TeleDoc-Doc-Medizingeräte wurden eingesetzt!** | **Wie gut sind Sie mit den <u>eingesetzten</u> Geräten zurechtgekommen? Vergeben Sie eine Note.** | |
|---|---|---|
| O Kamera | Kamera | |
| O Stethoskop | Stethoskop | |
| O Blutzuckergerät | Blutzuckergerät | |
| O Blutdruckgerät | Blutdruckgerät | |
| O Pocket WiWe EKG | Pocket WiWe EKG | |
| Patientenmonitor (PM) | Patientenmonitor (PM) | |
| O EKG über PM | - EKG über PM | |
| O Puls über PM | - Puls über PM | |
| O Blutdruck über PM | - Blutdruck über PM | |
| O Sauerstoffsättigung über PM | - Sauerstoffsättigung über PM | |
| O Temperatur über PM | - Temperatur über PM | |

**Was war beim Einsatz von TeleDoc-Geräten schwierig? Was wünschen Sie sich?**

# Einsatzprotokoll zur Televisite (8)

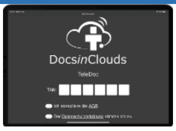


## Bewertung der TeleDoc-Software

**Wie gut sind Sie mit der TeleDoc-Software zu Recht gekommen?**
**Vergeben Sie eine Schulnote von 1 sehr gut bis 6 unzureichend.**

**Was war bei der Nutzung der TeleDoc-Software schwierig? Was wünschen Sie sich?**

## Einschätzung der ärztlichen Betreuung bei der Televisite

**Wie schätzen Sie die ärztliche Betreuung bei der durchgeführten Televisite ein?**
**Vergeben Sie eine Schulnote.**

| 1 | 2 | 3 | 4 | 5 | 6 |
|---|---|---|---|---|---|
| sehr gut | gut | befriedigend | ausreichend | mangelhaft | ungenügend |

**Was wäre für den konkreten Fall für ein bessere ärtzliche Versorgung per Televisite erforderlich bzw. wünschenswert?**

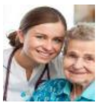

**Wie fand der bzw. die Bewohner/in die ärztliche Betreuung bei der Televiste?**
**Welche Schulnote wurden für die ärztliche Betreuung vergeben?**

**Tragen Sie bitte Anmerkungen oder Hinweise des Bewohners der Bewohnerin ein.**

# Einsatzprotokoll zur Televisite (9)

## Grunddaten zur Televisite

| Wann fand die Televisite statt? | Tag | | Monat | |
|---|---|---|---|---|
| | | | | |

Es handelte sich um …

- O … eine mit dem Arzt vorher geplante Televisite.
- O … eine aktuell erforderliche Televisite

**Aufgrund welcher Situation bzw. Beschwerden wurde die Televisite durchgeführt?**

……………………………………………………………………………………………………

**Was hätten Sie in dem konkreten Fall ohne Telemedizin unternommen?**

- O den Notruf 112 angerufen
- O den ärztlichen Bereitschaftsdienst angerufen
- O abgewartet, bis unser Arzt kommen kann

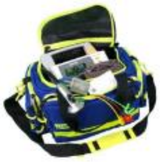

## Einsatz und Bewertung der TeleDoc-Medizingeräte

Bei der Televisite kamen …

- O <u>keine</u> TeleDoc-Medizingeräte
- O TeleDoc-Doc-Medizingeräte

zum Einsatz.

| Welche TeleDoc-Doc-Medizingeräte wurden eingesetzt! | Wie gut sind Sie mit den <u>eingesetzten</u> Geräten zurechtgekommen? Vergeben Sie eine Note. | |
|---|---|---|
| O Kamera | Kamera | |
| O Stethoskop | Stethoskop | |
| O Blutzuckergerät | Blutzuckergerät | |
| O Blutdruckgerät | Blutdruckgerät | |
| O Pocket WiWe EKG | Pocket WiWe EKG | |
| Patientenmonitor (PM) | Patientenmonitor (PM) | |
| O EKG über PM | - EKG über PM | |
| O Puls über PM | - Puls über PM | |
| O Blutdruck über PM | - Blutdruck über PM | |
| O Sauerstoffsättigung über PM | - Sauerstoffsättigung über PM | |
| O Temperatur über PM | - Temperatur über PM | |

**Was war beim Einsatz von TeleDoc-Geräten schwierig? Was wünschen Sie sich?**

# Einsatzprotokoll zur Televisite (9)

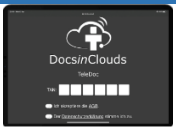


## Bewertung der TeleDoc-Software

**Wie gut sind Sie mit der TeleDoc-Software zu Recht gekommen?**
**Vergeben Sie eine Schulnote von 1 sehr gut bis 6 unzureichend.**

**Was war bei der Nutzung der TeleDoc-Software schwierig? Was wünschen Sie sich?**

## Einschätzung der ärztlichen Betreuung bei der Televisite

**Wie schätzen Sie die ärztliche Betreuung bei der durchgeführten Televisite ein?**
**Vergeben Sie eine Schulnote.**

| 1 | 2 | 3 | 4 | 5 | 6 |
|---|---|---|---|---|---|
| sehr gut | gut | befriedigend | ausreichend | mangelhaft | ungenügend |

**Was wäre für den konkreten Fall für ein bessere ärtzliche Versorgung per Televisite erforderlich bzw. wünschenswert?**

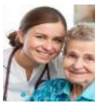

**Wie fand der bzw. die Bewohner/in die ärztliche Betreuung bei der Televiste?**
**Welche Schulnote wurden für die ärztliche Betreuung vergeben?**

**Tragen Sie bitte Anmerkungen oder Hinweise des Bewohners der Bewohnerin ein.**

# Einsatzprotokoll zur Televisite (10)

## Grunddaten zur Televisite

**Wann fand die Televisite statt?**

| Tag | | Monat | |
|---|---|---|---|
| | | | |

**Es handelte sich um …**

O … eine mit dem Arzt vorher geplante Televisite.
O … eine aktuell erforderliche Televisite

**Aufgrund welcher Situation bzw. Beschwerden wurde die Televisite durchgeführt?**

……………………………………………………………………………………………………

**Was hätten Sie in dem konkreten Fall ohne Telemedizin unternommen?**

O den Notruf 112 angerufen
O den ärztlichen Bereitschaftsdienst angerufen
O abgewartet, bis unser Arzt kommen kann

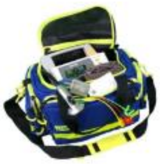

## Einsatz und Bewertung der TeleDoc-Medizingeräte

**Bei der Televisite kamen …**

O <u>keine</u> TeleDoc-Medizingeräte
O TeleDoc-Doc-Medizingeräte

**zum Einsatz.**

| **Welche TeleDoc-Doc-Medizingeräte wurden eingesetzt!** | **Wie gut sind Sie mit den <u>eingesetzten</u> Geräten zurechtgekommen? Vergeben Sie eine Note.** | |
|---|---|---|
| O Kamera | Kamera | |
| O Stethoskop | Stethoskop | |
| O Blutzuckergerät | Blutzuckergerät | |
| O Blutdruckgerät | Blutdruckgerät | |
| O Pocket WiWe EKG | Pocket WiWe EKG | |
| Patientenmonitor (PM) | Patientenmonitor (PM) | |
| O EKG über PM | - EKG über PM | |
| O Puls über PM | - Puls über PM | |
| O Blutdruck über PM | - Blutdruck über PM | |
| O Sauerstoffsättigung über PM | - Sauerstoffsättigung über PM | |
| O Temperatur über PM | - Temperatur über PM | |

**Was war beim Einsatz von TeleDoc-Geräten schwierig? Was wünschen Sie sich?**

# Einsatzprotokoll zur Televisite (10)

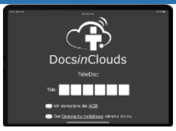


## Bewertung der TeleDoc-Software

**Wie gut sind Sie mit der TeleDoc-Software zu Recht gekommen?**
**Vergeben Sie eine Schulnote von 1 sehr gut bis 6 unzureichend.** ☐

**Was war bei der Nutzung der TeleDoc-Software schwierig? Was wünschen Sie sich?**

## Einschätzung der ärztlichen Betreuung bei der Televisite

**Wie schätzen Sie die ärztliche Betreuung bei der durchgeführten Televisite ein?**
**Vergeben Sie eine Schulnote.**

| 1 | 2 | 3 | 4 | 5 | 6 |
|---|---|---|---|---|---|
| sehr gut | gut | befriedigend | ausreichend | mangelhaft | ungenügend |

☐

**Was wäre für den konkreten Fall für ein bessere ärtzliche Versorgung per Televisite erforderlich bzw. wünschenswert?**

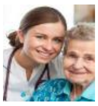

**Wie fand der bzw. die Bewohner/in die ärztliche Betreuung bei der Televiste?**
**Welche Schulnote wurden für die ärztliche Betreuung vergeben?** ☐

**Tragen Sie bitte Anmerkungen oder Hinweise des Bewohners der Bewohnerin ein.**

# Platz für weitere Kommentare

**Bitte bei den Kommentaren das Televisiten-Datum vermerken.!**

# Fragen an die Bewohner/innen

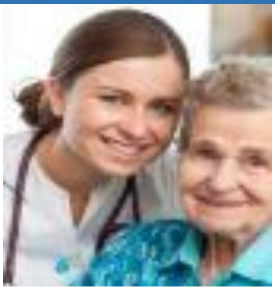

**Bitte fragen Sie den Bewohner bzw. die Bewohnerin möglichst unmittelbar nach der Televisite:**

**3) „Wie fanden Sie die ärztliche Betreuung bei der Televiste?“**

Platz für Klebezettel-Notizen!

**4) Welche Schulnote vergeben sie als Patient für die ärztliche Betreuung bei der Televisite?**

Sagen Sie, was welche Note bedeutet und zeigen Sie ggf. die Schulnoten mit den Smilies.

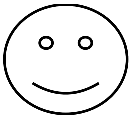

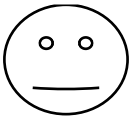

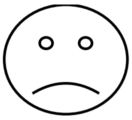

| 1 | 2 | 3 | 4 | 5 | 6 |
|---|---|---|---|---|---|
| sehr gut | gut | befriedigend | ausreichend | mangelhaft | ungenügend |

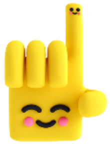

**Tragen Sie die Anmerkungen sowie die Schulnote jeweils am Ende des Einsatzprokolls ein!**

# AIDA

## Arbeitsentwicklung In Der Altenpflege

**Informationen zum Projekt unter www.projekt-aida.org**

| | |
|---|---|
| Logo eines Projektpart-ners | Logo eines Projektpart-ners |
| Logo eines Projektpart-ners | Logo eines Projektpart-ners |
| Logo eines Projektpart-ners | Logo eines Projektpart-ners |
| Logo eines Projektpart-ners | Logo eines Projektpart-ners |

EUROPÄISCHE UNION
Investition in unsere Zukunft
Europäischer Fonds
für regionale Entwicklung

Die Landesregierung
Nordrhein-Westfalen

Zukunft der Altenpflege
