# Supplementary material for: Long-Term Adoption of Televisits in Nursing Homes During the COVID-19 Crisis and Following Up Into the Postpandemic Setting: Mixed Methods Study
Source: JMIR Aging. 2024 Jun 6;7:e55471. doi: 10.2196/55471 (PMC11190630; doi:10.2196/55471)
Supplement: Multimedia Appendix 4 [file aging_v7i1e55471_app4.pdf]

ENGLISH VERSION (TRANSLATED FROM GERMAN)

ORIGINAL GERMAN VERSION BELOW

Questions to the nursing staff

**Interview partners:** Nurses having performed televisits in stationary geriatric care within the "AIDA" project (Arbeitsentwicklung in der Altenpflege) funded by the European Regional Development Fund (ERDF) 2014-2020 of the European Union and by the State of North Rhine-Westphalia, Germany

Structure:

1. Part 1: evaluation of the expectations and experiences with televisits, overall assessment
2. Part 2: impact of the implementation of televisits
3. Part 3: televisits across the project period
4. Part 4: perspective/ outlook on telemedicine in stationary geriatric care

Definitions/ Abbreviations :

televisits = video consultations with a physician, where (a) patients (here the residents) are accompanied by non-physician healthcare professionals and (b) additional point-of-care diagnostic devices are available to be used on the patients (here the residents) in order to measure vital signs and perform clinical examination (e.g. via a stethoscope)
TeleDoc = commercially available telemedicine system for performing televisits, distributed by Docs In Clouds Telecare GmbH
NH1 = nursing home 1 cooperating in the project
NH2 = nursing home2 cooperating within the AIDA-project
OOH-GP-service = out-of-hours GP-service for medical presentations needing prompt medical assessment and that cannot be postponed to the following day. The OOH-GP-service however excludes emergency situations needing to call the ambulance or going into the emergency department, organised in Germany by the local organisation of office-based physicians („Kassenärztliche Vereinigung")

b) Interview

NH1: Hello. You are a nurse here in NH1 and I am happy to do a short interview with you about telemedicine. Your institution has been using the TeleDoc-system within the AIDA project very regularly for more than two years, with televisits taking place almost every week.

NH2: You are a nurse here at NH2, and I am pleased to conduct a brief interview with you about telemedicine. Your institution used the TeleDoc-system during the third and last COVID-19 lockdown in Germany.

During these televisits, the residents undergo a medical examination, receive a medical assessment and, if necessary, receive therapeutic measures. The GP is connected to the NH out of her practice via

a telemedical interface. You are the nurse next to the resident and assist the remote working GP while having access to point-of-care medical diagnostic devices connected to TeleDoc. I would like to invite you to take about 30 minutes for an interview. The aim is to briefly discuss your experience with televisits using the TeleDoc. We want to collect the experiences of several nurses using the TeleDoc and to evaluate them scientifically. The focus will on the impact/ the benefits of televisits for you as a nurse and how you deal with the innovation of 'telemedicine'. The benefits for the residents have been evaluated separately and are explicitly not the subject of this conversation.

Part 1: evaluation of the expectations and experiences with televisits, overall assessment

At first, I have a few open questions regarding the general use of telemedicine. Most nurses had not used telemedicine before, as it is an entirely innovative concept in stationary elderly care. In Germany, this usage is not yet widely spread.

What expectations did you have regarding telemedicine and televisits.
Relating to your profession?<br>*In relation to the residents?*<br>*Did you have specific expectations regarding the technology?*
What experiences did you gain with televisits?<br><br>*Do you want to describe, how you dealt with them? Regarding the handling/ the technology?*<br>*How did you integrate them in your working routine? How was the interaction with the residents? How did it work out for you?*
How would you rate the overall experience?

Part 2: impact of the implementation of televisits

Introducing telemedicine, as was done in the AIDA project, changes your daily work routine as a nurse. In the AIDA project, we have developed solutions on how the work processes in stationary elderly care can be structured so that the innovation of 'telemedicine' benefits not only the residents (through better care) but also you as a nurse. To this, we have a few questions.

(1) The televisits with the coordinating facility GP are designed to provide you with an easy way to get help with residents' medical issues. Especially in moments, during both routine and unscheduled visits, in which you feel unsure of what to do how to proceed. Questions such as 'Should I call the GP now, or can it wait until the next facility visit?' or 'The situation may be more urgent, I need to clarify this quickly?" "Do I need to call the emergency services?"

Many nurses describe experiencing psychological distress in these situations. How did, and do, you feel in such moments?

Uncertainty in assessing the situation? Was this psychological pressure particularly strong during the COVID-19 lockdown?
Do you feel that the availability of telemedicine has reduced this psychological burden? Because you can now reach the doctor more easily and often? (NH1: because you know that telemedical visits take place every Wednesday and/or the doctor can be reached for a televisit during office hours)."
Is there simply more frequent contact now? Do you feel more confident in seeking help?
If yes, has the psychological stress<br>- slightly-<br>- noticeably-<br>or<br>- very noticeably<br>decreased?
Do you have specific examples where telemedical visits have relieved or are relieving you of this psychological pressure?

(2) Telemedicine eliminates travel distances. This reduces the time expenditure for physicians (GPs and specialists). Additionally, telemedicine can also save time for caregivers, especially in unscheduled “emergency” situations, when calling doctors and the out-of-hours GP service is avoided. In such situations, there are often long call queues or multiple calls back-and-forth between the practice and the facility before contact is made.

Do you feel that televisits save time for you?
Or could be timesaving?
Do the physicians save time?

(3) In TeleDoc, medical prescriptions and orders are documented and time-stamped, providing a clear record. In the day-to-day practice of inpatient elderly care, however, many orders are still given verbally over the phone.

How do you assess the aspect of improved legal certainty, with orders being clearly documented and signed by the doctors with a timestamp

(4) NH2 (additional explanation): In the other NH, routine televisits have been performed almost weekly since 2021.

NH1 + NH2: Our evaluation of hospital admissions, as well as other studies in the field of telemedicine, suggest that with regular use, there is a learning effect in the area of medical skills for nurses.

Do you feel that your medical-clinical skills have improved through regular telemedical visits? For example, do you recognize changes in the residents' conditions earlier and can you assess them better?<br>How does this come about? What do you think? What have you personally taken away from it?
You use medical devices to take vital signs before visits, to “prepare” for visits. Does this elevate your role as healthcare professional?<br>*Do you sometimes become aware of certain things while taking measurements?*
Using the TeleDoc and performing televisits had to be learned at the beginning. What specific challenges did you face initially?<br>*Aims to get the personal experiences of the nurses. What was particularly challenging for them? Pre- and post-COVID, the synchronous/asynchronous visits…*
NH1 The training process was challenging and lengthy due to COVID restrictions and high levels of sickness absence. Over the course of the project, we transitioned from group to individual training, especially helped by the medical student. We have now developed a comprehensive training concept with simulated televisits, that is implemented in a tandem approach. What do you think of this training concept?<br><br>NH2: The training process was challenging and lengthy due to COVID restrictions and high levels of sickness absence. Over the course of the project, we transitioned from group to individual training, especially helped by the medical student in the other NH. We have now developed a comprehensive training concept with simulated televisits, that is then implemented in a tandem approach. What do you think of this training concept?<br><br>*Two levels (1) simulated televisit (2) tandem format.*<br>*General evaluation of the training concept?*

(5) The implementation of televisits changed the collaboration between the nursing home and the medical practice.

From your perspective, has the communication and collaboration with the GP changed?<br>Is the collaboration now more collegial and easier? Do you approach the GPs earlier before the situation deteriorates?
How challenging is communication for you during televisits with the TeleDoc, in this sort of "triangular relationship" between the resident, the doctor, and yourself?<br>*Is it unfamiliar? a matter of practice? What changes there? That everyone feels valued/ that residents can be included. How do you involve the residents in the process?*

Part 2: televisits across the project period

The project has gone through many phases, and both the technology and the work processes have been iteratively adapted. We would also like to get your views on this. We have prepared some statements for this purpose and ask you to comment on them.

(6) From an external point of view, it seems that televisits have been evaluated differently across the project period and its different phases of adaptation.

During the COVID lockdown, the benefits of telemedicine were immediately apparent to us and seen as a positive development. Please comment on this statement.
With the lifting of contact restrictions, the benefits seemed less tangible. Please comment on this statement.
NH1; Afterwards, the benefit became apparent only after many repeated adaptations and trainings. Please comment on this statement

NH2: As a result, many barriers became apparent. The visits were not time efficient and not well integrated into the work processes. Please comment on this statement. Do you agree or disagree? The GP then preferred home visits again. Why did the long-term implementation fail?
Balance of (nursing) effort/ benefit
NH1: The asynchronous visits, the transition to the “virtual waiting room”, and the adjustment of the training concept were the main adaptations during the project. In your opinion, what particularly contributed to the success of the sustainable practice implementation?"<br><br>NH2: The experiences in your NH, as well as those from the other NH, have significantly contributed to optimizing the system and workflows. The software has been modified. It now allows to edit and prepare multiple visits at the same time, so that measurements of several residents can be taken before the visit. Workflows have also been changed. Do you feel that this has improved the system? What do you expect from these changes?
Is there a change that you have noticed the most?
How was the feedback from the residents?

Part 4: perspective/ outlook on telemedicine in stationary geriatric care

(7) Due to the positive experiences and excellent results, additional NH will be equipped with the TeleDoc system. Soon, other nurses will be working with the system.

What advice would you five to these nurses?

(8) Telemedicine and televisits in stationary geriatric care

… described in three words?

GERMAN VERSION

Interviewfragen an die Pflegekräfte

Zielgruppe: Pflegefachkräfte, die Televisiten in der stationären Altenpflege im Rahmen des EFRE geförderten (EU und Land NRW) Projektes AIDA (Arbeitsentwicklung in der Altenpflege) umgesetzt haben

Aufbau:

5. Teil 1: offene Fragen zu Erwartungen/ Erfahrungen und Bewertung der Televisiten
6. Teil 2: Auswirkungen der Implementation von Televisiten
7. Teil 3: Televisiten über die Projektlaufzeit
8. Teil 4: Perspektiven der Televisiten in der stationären Altenpflege

Definitionen/ Abkürzungen:
Televisiten = Videosprechstunden, bei denen (a) die Patienten (hier die Bewohnenden) von nicht ärztlichen Fachpersonal begleitet werden und (b) zusätzliche Point-of-Care-Diagnostikgeräte auf Seite der Patienten (hier die Bewohnenden) zur Messung von Vitalparametern und zur Durchführung der klinischen Untersuchung (z.B. Stethoskop) zur Verfügung stehen
TeleDoc = kommerziell verfügbares telemedizinisches Gesamtsystem zur Durchführung von Televisiten, vertrieben von der Docs In Clouds Telecare GmbH
PH1 = Pflegeheim 1 des Projektes
PH2 = Pflegeheim 2 des Projektes
KV = Kassenärztliche Vereinigung, stellt den KV-Notdienst über die 116 117: ärztlicher Bereitschaftsdienst außerhalb der Sprechstundenzeiten der niedergelassenen Ärzte für Situationen, in denen man nicht bis zum nächsten Tag warten kann, aber keine Notfallsituation im Sinne von einer Alarmierung der 112 und oder der Notwendigkeit eines Besuches der Notaufnahme besteht.

b) Interview

PH1: Hallo. Sie sind Pflegekraft hier im PH1 und ich freue mich, dass ich mit Ihnen ein kurzes Interview zur Telemedizin machen kann. In Ihrer Einrichtung wird im Rahmen des Projektes AIDA der TeleDoc seit mittlerweile über zwei Jahren sehr regelmäßig genutzt, nahezu wöchentlich finden Televisiten statt.

PH2: Hallo. Sie sind Pflegekraft hier im PH2 und ich freue mich, dass ich mit Ihnen ein kurzes Interview zur Telemedizin machen kann. In Ihrer Einrichtung wurde im Rahmen des Projektes AIDA der TeleDoc vor allem während der Bundesnotbremse 2021 („3. Coronalockdown“) genutzt.

In diesen Televisiten finden für die Bewohnenden des Heimes eine ärztliche Untersuchung, eine ärztliche Beurteilung und gegebenenfalls therapeutische Maßnahmen statt. Die Ärztin ist aus der Praxis telemedizinisch angebunden, und sie betreuen die Bewohnenden vor Ort und assistieren der Ärztin aus der Ferne, wozu sie über zusätzliche medizinische Geräte am TeleDoc verfügen. Gerne möchte ich Sie bitten, sich für ein circa 30-minütiges Interview Zeit mit mir zu nehmen. Das Ziel ist es hierbei, kurz und bündig über Ihre Erfahrungen von Televisiten mit dem TeleDoc zu sprechen. Wir

möchten Erfahrungen von mehreren Anwendern der Telemedizin sammeln und diese wissenschaftlich auswerten. Der Fokus liegt hier auf den Auswirkungen/dem Nutzen der Telemedizin für Sie als Pflegefachkraft und ihrem Umgang mit der Innovation „Telemedizin“. Den Nutzen für die Bewohnenden haben wir separat ausgewertet und ist hier explizit nicht Gegenstand des Gespräches.

<u>Teil1: offene Fragen zu Erwartungen/ Erfahrungen und Bewertung der Telemedizin</u>

Zunächst habe ich ein paar offene Fragen zur Nutzung der Telemedizin allgemein. Die meisten Pflegekräfte hatten vorher noch keine Telemedizin genutzt, denn Telemedizin ist in der stationären Altenpflege ist ein absolut innovatives Konzept. In Deutschland ist diese Nutzung daher noch nicht weit verbreitet.

Welche Erwartungen bestanden bei Ihnen in Bezug auf die Telemedizin?
Dem Beruf gegenüber?<br>*Den Bewohnenden gegenüber?*<br>*Hatten Sie besondere Erwartungen, was die Technik anbetrifft?*
Welche Erfahrungen haben sie mit den Televisiten gesammelt?<br><br>*Möchte Sie beschreiben, wie Sie damit umgegangen sind? Vom Handling / der technischen Seite?*<br>*Wie haben Sie das in den Arbeitsalltag integriert? Wie war der Umgang mit den Bewohnern? Wie hat das für Sie funktioniert?*
Wie fällt die Gesamtbewertung aus?

<u>Teil 2: Auswirkungen der Implementation von Televisiten</u>

Die Einführung von Telemedizin, wie im Projekt AIDA geschehen, verändert Ihren täglichen Arbeitsalltag als Pflegefachkraft. In AIDA haben wir Lösungen erarbeitet, wie die Arbeitsprozesse in der stationären Altenpflege so gestaltet werden können, dass die Innovation „Telemedizin“ nicht nur den Bewohnenden (durch eine bessere Versorgung), sondern auch Ihnen als Pflegefachkraft zugutekommt. Hierzu haben wir zunächst einige Fragen.

(1) Die Televisiten mit der koordinierenden Heimärztin sollen für Sie eine Möglichkeit sein, um sich bei *medizinischen Fragen* zu den Bewohnenden möglichst unkompliziert Hilfe holen zu können. Konkret geht es sowohl bei den Routinevisiten als auch bei den außerplanmäßigen Visiten um Momente, bei denen sie sich unsicher fühlen und nicht genau wissen, wie Sie sich zu verhalten haben. „Muss ich jetzt den Hausarzt anrufen, oder kann ich damit bis zur nächsten Heimvisite warten?“ oder „Die Situation ist vielleicht doch akuter, das sollte ich jetzt schnell abklären. Muss ich hierfür den ärztlichen Notdienst anrufen?“

Viele Pflegefachkräfte beschreiben einen psychischen Druck in diesen Situationen. Wie erging und ergeht es Ihnen in solchen Situationen?
 <br><br>*Unsicherheit, die Situation einzuschätzen?*<br>*War dieser psychische Druck besonders stark während des Coronalockdowns?*
Haben Sie das Gefühl, dass dieser psychische Druck durch das Angebot der Televisiten jetzt niedriger ist? Weil sie nun niederschwelliger und häufiger die Ärztin erreichen? (PH1: weil sie wissen, dass jeden Mittwoch Televisiten stattfinden und/oder die Ärztin auch während der Sprechstunde für eine Televisite erreicht werden kann)
 <br>*Besteht jetzt einfach häufiger ein Kontakt? Traut man sich mehr Hilfe zu suchen?*
Falls ja, hat der psychische Druck<br>- Leicht-<br>- Deutlich-<br>oder<br>- Sehr deutlich<br>abgenommen?
Haben Sie konkrete Beispiele, in denen Sie die Televisiten von diesem psychischen Druck entlastet hat oder entlastet?

(2) Durch die Telemedizin fallen Anfahrtswege weg. Damit sinkt auch der Zeitaufwand für die Ärzte (sowohl für Hausärzte als auch Ärzte der KV). Zudem kann die Telemedizin auch eine Zeitersparnis für die Pflegekräfte darstellen. Dies gilt insbesondere in den Notfallsituationen, wenn der Anruf bei den Ärzten und beim KV-Notdienst wegfällt. Dort fallen oftmals lange Wartezeiten in den Warteschleifen an oder es wird mehrmals zwischen Praxis und Einrichtung hin- und hertelefoniert, bis man sich erreicht.

Haben Sie das Gefühl, dass Televisiten für Sie eine Zeitersparnis bedeutet?
 Oder bedeuten kann
Besteht eine Zeitersparnis für Ärzte?

(3) In dem TeleDoc werden Anordnungen der Ärzte mit einem Zeitstempel dokumentiert festgehalten und sind somit eindeutig hinterlegt. In der täglichen Praxis in der stationären Altenpflege werden hingegen noch viele Anordnung telefonisch gemacht.

Wie bewerten sie den Aspekt der besseren Rechtssicherheit, dass Anordnungen klar dokumentiert und mit dem Zeitstempel durch die Ärzte signiert sind?

(4) PH2(zusätzliche Erklärung): In dem anderen Pflegeheim finden seit 2021 nahezu wöchentlich routinemäßige Televisiten statt.

PH1 + PH2: Unsere Auswertungen zu den Krankenhauseinweisungen, als auch andere Studien im Bereich der Telemedizin legen nahe, dass mit der routinemäßigen Anwendung ein Lerneffekt auch im Bereich der medizinischen Fähigkeiten für die Pflegekräfte eintritt.

Haben Sie das Gefühl, dass sich ihre medizinisch-klinischen Fähigkeiten durch die regelmäßigen Televisiten verbessert haben? Erkennen Sie beispielsweise Zustandsveränderungen bei den Bewohnenden früher und können diese besser einschätzen?<br>Wie kommt der zustande? Was denken Sie? Was haben Sie persönlich mitgenommen
Sie nutzen die Medizingeräte, um die Vitalparameter bereits vor den Visiten zu messen, und so die „Visiten vorzubereiten“. Ist das auch hier für Sie eine Aufwertung in ihrer Rolle als Pflegefachkraft?<br>Werden einem manchmal beim Messen bereits gewisse Dinge bewusst?
Die Nutzung des TeleDoc und die Umsetzung von Televisiten musste ja zunächst gelernt werden. Was waren für Sie die besonderen Herausforderungen am Anfang?<br>*Ziel auf die persönlichen Erfahrungen der Mitarbeitenden zu kommen. Was war für sie besonders herausfordernd? Während und nach Corona, die synchronen/ asynchronen Visiten etc*
PH1: Der Schulungsprozess war durch die Coronarestriktionen und den hohen Krankenstand sehr schwer und langwierig. Wir sind im Verlaufe des Projektes von Gruppen- zu Einzelschulungen, insbesondere mit dem Medizinfamulanten übergegangen. Mittlerweile haben wir ein ganzes Schulungskonzept mit simulierten Einsatzszenarien entwickelt, was dann im Tandemkonzept umgesetzt wird. Was halten Sie von diesen Schulungskonzept?<br><br>PH2: Der Schulungsprozess war durch die Coronarestriktionen und den hohen Krankenstand sehr schwer und langwierig. Wir sind im Verlaufe des Projektes von Gruppen- zu Einzelschulungen, insbesondere mit einem Famulanten in dem anderen Heim übergegangen. Mittlerweile haben wir ein ganzes Schulungskonzept mit simulierten Einsatzszenarien entwickelt, was dann im Tandemkonzept umgesetzt wird. Was halten Sie von diesen Schulungskonzept?

Zwei Ebenen (1) Szenarien (2) Tandemformat.<br>*Generelle Bewertung des Schulungskonzeptes*

(5) Im Rahmen der Einführung der Televisiten hat sich die Zusammenarbeit zwischen dem Pflegeheim und der Praxis geändert.

Haben sich aus Ihrer Sicht die Kommunikation und Zusammenarbeit mit der Hausärztin geändert?<br>Ist die Zusammenarbeit jetzt kollegialer, einfacher? Traut man sich jetzt „früher“ auf die Hausärzte zu zugehen, bevor sich die Situation verschlechtert?
Wie herausfordernd ist für sie die Kommunikation über den TeleDoc, in so einer Art „Dreiecksbeziehung“ zwischen dem/der Bewohner/-in, der Ärztin und Ihnen?<br>Ungewohnt? Übungssache? Was verändert sich da?<br>Wie binden Sie die Bewohner ein?

Teil 2: Televisiten über die Projektlaufzeit

Das Projekt hat viele Phasen durchlebt, und die Technik, sowie die Arbeitsprozesse sind wiederholt angepasst worden. Auch hierzu möchten wir gerne Ihre Sicht der Dinge einholen. Hierzu haben wir einige Aussagen vorbereitet, wir bitten Sie diese zu kommentieren

(6) Von außen betrachtet schien es, als ob die Telemedizin im Laufe des Projektes und den unterschiedlichen Anpassungsphasen unterschiedlich bewertet wurde.

Während des Coronalockdowns war der Nutzen der Telemedizin für uns direkt ersichtlich und eine gute Sache. Kommentieren Sie diese Aussage.
Mit Aufhebung der Kontaktbeschränkungen war der Nutzen zunächst weniger greifbar Kommentieren Sie diese Aussage.

PH1: : Anschließend zeigte sich der Nutzen erst nach vielen wiederholten Anpassungen und Schulungen. Kommentieren Sie diese Aussage.

PH2: Anschließend zeigten sich die viele Barrieren. Die Visiten waren nicht zeiteffizient und nicht gut in die Arbeitsprozesse integriert. Kommentieren Sie diese Aussage. Stimmen Sie oder nicht zu? Die Hausärztin präferierte dann wieder Hausbesuche. Warum scheiterte die nachhaltige Praxisimplementierung?

Balance (pflegerischer) Aufwand/ Nutzen

PH1: Die asynchronen Visiten, die Umstellung auf „das virtuelle Wartezimmer“ und die Anpassung des Schulungskonzeptes waren die großen Anpassungen während der Projektlaufzeit. Was hat Ihrer Meinung nach besonders zum Gelingen der nachhaltigen Praxiseinführung beigetragen?

PH2: Die Erfahrungen aus ihrem Pflegeheim haben ebenso wie die Erfahrungen aus dem anderen Heim maßgeblich dazu beigetragen, das System und die Arbeitsprozesse zu optimieren. Die Software wurde verändert, um mehrere Visiten gleichzeitig bearbeiten und „vorbereiten“ zu können, d.h. dass man vor der Visite bereits die Messungen für mehrere Bewohner machen kann. Die Arbeitsprozesse wurden ebenfalls geändert. Halten Sie das System so für verbessert? Was versprechen Sie sich hiervon?

Gibt es eine Veränderung, die sie am stärksten gemerkt haben?

Wie bewerten Sie die Resonanz der Bewohner?

Teil 4: Perspektiven der Telemedizin in der stationären Altenpflege

(7) Aufgrund der guten Erfahrungen und der sehr guten Ergebnisse werden weitere Heime mit dem TeleDoc-System ausgestattet. Bald werden andere Pflegekräfte mit dem System arbeiten.

Was würden Sie denen gerne mit auf den Weg geben?

(8) Telemedizin und Televisiten in der stationären Altenpflege …

… bedeuten für mich in drei Worten:
