# Supplementary material for: Long-Term Adoption of Televisits in Nursing Homes During the COVID-19 Crisis and Following Up Into the Postpandemic Setting: Mixed Methods Study
Source: JMIR Aging. 2024 Jun 6;7:e55471. doi: 10.2196/55471 (PMC11190630; doi:10.2196/55471)
Supplement: Multimedia Appendix 5 [file aging_v7i1e55471_app5.pdf]

ENGLISH VERSION (TRANSLATED FROM GERMAN)

ORIGINAL GERMAN VERSION BELOW

Follow-up questionnaire

AIDA

Question 1: How often did you perform televisits with residents?

- ◯ Never
- ◯ 1-5 times
- ◯ 5-10 times
- ◯ More than 10 times

Question 2: How do you asses the necessity or priority to use televisits (with transmissions of PoC- data) for the medical care of NH residents?

	Very high priority	high priority	lower priority	unnecessary
with General Physicians	◯	◯	◯	◯
with out-of-hours GPs/ with emergency medical physicians	◯	◯	◯	◯
with neurologists/ psychiatrists	◯	◯	◯	◯
with dermatologists	◯	◯	◯	◯
with specialists in internal medicine	◯	◯	◯	◯

Question3: with other specialists, namely... (indicate also the priority level)

[ ]

Questions 4 bis 7: To what extent do you agree with the following statements?

Question 4: Televisits are entirely suitable for examination of the residents and initiating treatment decisions in case of GP-related medical queries

- ○ Full approval
- ○ Predominant approval
- ○ Mild disagreement
- ○ Full disagreement

Question 5: What is the impact of televisits on the residents' care?

- ○ better medical care
- ○ Neutral effect on the medical care
- ○ Lower medical care

Question 6: Performing televisits is difficult in our residents

- ○ Full approval
- ○ Predominant approval
- ○ Mild disagreement
- ○ Full disagreement

Question 7: Televisits should only be performed exceptionnaly.

- Full approval
- Predominant approval
- Mild disagreement
- Full disagreement

Question 8: Which groups of residents or patients do particularly benefit from televisits?

Question 9: For which groups of residents or patients are teleconsultations less or not suitable?

Question 10: As a nursing professional, how do you rate the physician's supervision during televisits. Please assign a grade from 1 to 6..

Very good deficient

1	2	3	4	5	6

Questions 11 bis 13: To what extent do you agree with the following statements?

Question 11: The TeleDoc-software is user-friendly.

- Full approval
- Predominant approval
- Mild disagreeement
- Full disagreement

Question 12: The point-of-care diagnostic devices of the Tele-Doc system are user-friendly.

- ◯ Full approval
- ◯ Predominant approval
- ◯ Mild disagreeemnt
- ◯ Full disagreement

Question 13: Taking account of the user feedback allowed to achieve improved user-friendliness of the whole TeleDoc-system for stationary care.

- ◯ Full approval
- ◯ Predominant approval
- ◯ Mild disagreement
- ◯ Full disagreeement

Question 14: Which changes or measures could further improve the user-friendliness?

[ ]

Question 15: How do you assess the workload of televisits regarding time expenditure (considering the time spent before, during, and after the session) in comparison to the previous practice of in-person primary care consultations?

	timesaving	no changes in time expenditure	time-consuming
for scheduled routine visits	◯	◯	◯
for unscheduled GP visits	◯	◯	◯

Question 16: What impacts have you observed following the introduction of the TeleDoc system and the implementation of televisits?

(Multiple Choice Question: please select all answers that apply!)

- [ ] Empowered participation and appreciation through superiors and/ or team
- [ ] Additional burden due to digital/ technological stress (for example due to incertainty releative to stability/ functionality oft the technology or relative to correct use/ handling of the technology)
- [ ] Reduced burden through the resolution of medical questions, improved ability to act and higher legal certainty
- [ ] Improved software skills
- [ ] Improved skills in the use of medical diagnostic devices
- [ ] Improved clinical skills
- [ ] Increased recognition of the competencies as nurse by the GP
- [ ] Increased recognition of the competencies as nurse by the residents
- [ ] Improved communication with the GP

ORIGINAL GERMAN VERSION

Abschlusserhebung

AIDA

Anmerkungen: Televisiten und Telekonsultation werden synonym gebraucht

Frage 1: Wie oft haben Sie Televisiten mit Bewohnern durchgeführt?

- ◯ Nie
- ◯ 1-5 mal
- ◯ 5-10 mal
- ◯ mehr als 10 mal

Frage2: Wie schätzen Sie die Notwendigkeit bzw. Dringlichkeit zur Nutzung von Televisiten bzw. Telekonsultationen (Visiten mit Diagnoseübertragung aus der Ferne) für die medizinische Versorgung von Plegeheimbewohner*innen ein?

	sehr dringlich	dringlich	wenig dringlich	unnötig
mit Hausärzten	◯	◯	◯	◯
mit Bereitschafts-/Notärzten	◯	◯	◯	◯
mit Neurologen/Psychiatern	◯	◯	◯	◯
mit Dermatologen	◯	◯	◯	◯
mit Internisten	◯	◯	◯	◯

Frage3: mit anderen Fachärzten, und zwar... (schreiben Sie auch bitte die Dringlichkeit dazu)

[ ]

Fragen 4 bis 7: Inwieweit stimmen Sie folgenden Aussagen zu?

Frage 4: Mit dem Teledoc-System können gesundheitliche Probleme von Pflegeheimbewohnern für Behandlungsentscheidungen durch Hausärzte ausreichend diagnostiziert werden?

- ◯ stimmt voll und ganz
- ◯ stimmt überwiegend
- ◯ stimmt eher nicht
- ◯ stimmt überhaupt nicht

Frage 5: Welche Auswirkungen hat die Nutzung von Telekonsultationen für die hausärztliche Versorgung der Pflegebedürftigen?

- ◯ bessere ärztliche Versorgung
- ◯ gleichbleibende ärztliche Versorgung
- ◯ schlechtere ärztliche Versorgung

Frage 6: Televisiten sind bei unseren Bewohnern schwierig

- ◯ stimmt voll und ganz
- ◯ stimmt überwiegend
- ◯ stimmt eher nicht
- ◯ stimmt überhaupt nicht

Frage 7: Televisiten sollten nur im absoluten Ausnahmefall genutzt werden

- ◯ stimmt voll und ganz
- ◯ stimmt überwiegend
- ◯ stimmt eher nicht
- ◯ stimmt überhaupt nicht

Frage 8: Für welche Bewohner- bzw. Patientengruppen hat die Nutzung von Telekonsultationen besondere Vorteile?

Frage 9: Für welche Bewohner- bzw. Patientengruppen sind Telekonsultationen weniger oder nicht geeignet?

Frage 10: Wie schätzen Sie als Pflegekraft die ärztliche Betreuung bei Telekonsultationen ein. Vergeben Sie eine Schulnote von 1 bis 6.

sehr gut ungenügend

1	2	3	4	5	6

Fragen 11 bis 13: Inwieweit stimmen Sie folgenden Aussagen zur Nutzerfreundlichkeit zu?

Frage 11: Die TeleDoc-Software ist nutzerfreundlich gestaltet.

- ◯ stimmt voll und ganz
- ◯ stimmt überwiegend
- ◯ stimmt eher nicht
- ◯ stimmt überhaupt nicht

Frage 12: Die Diagnosegeräte des TeleDoc-Systems sind nutzerfreundlich.

- ○ stimmt voll und ganz
- ○ stimmt überwiegend
- ○ stimmt eher nicht
- ○ stimmt überhaupt nicht

Frage 13: Im AIDA-Projekt wurde durch Umsetzung von Hinweisen der Anwender*innen eine bessere Nutzerfreundlichkeit des gesamten TeleDoc-Systems für die stationäre Pflege erzielt.

- ○ stimmt voll und ganz
- ○ stimmt überwiegend
- ○ stimmt eher nicht
- ○ stimmt überhaupt nicht

Frage 14: Durch welche Änderungen bzw. Maßnahmen könnte die Nutzerfreundlichkeit noch verbessert werden?

Frage 15: Wie beurteilen Sie den zeitlichen Aufwand von Telekonsultationen (unter Berücksichtigung Aufwandes vor, während und nach der Durchführung) in Verhältnis zu der bisherigen Praxis hausärztlichen Konsultationen.

	zeitlich weniger aufwendig	zeitlich gleich aufwendig	aufwendiger
bei der Durchführung von hausärztlichen Routinevisiten	○	○	○
für außerplanmäßige hausärztliche Konsultationen	○	○	○

Frage 16: Welche Auswirkungen haben Sie durch die Einführung des TeleDoc-Systems und Nutzung von Telekonsultationen wahrgenommen?

(Mehrfachantworten: Alles Zutreffende bitte ankreuzen!)

- [ ] Einbringen persönliche Stärken und Wertschätzung durch Vorgesetzte und/oder Team
- [ ] zusätzliche Belastung durch digitalen/technischen Stress (z.B. durch Unsicherheit in Hinblick auf Stabilität/Funktionalität der Technik oder korrekte Handhabung Technik)
- [ ] Kompetenzzuwachs in Hinblick auf Softwarenutzung
- [ ] Entlastung durch Klärung ärztlicher Fragen und bessere Handlungs- und Rechtsicherheit
- [ ] Kompetenzzuwachs beim Einsatz medizinisch-technischer Geräte
- [ ] Kompetenzzuwachs in Hinblick auf medizinische Fragestellungen
- [ ] Stärkere Wahrnehmung der Kompetenzen als Pflegekraft von Bewohnerseite
- [ ] Verbesserung der Kommunikation mit Hausarzt
- [ ] Stärkere Wahrnehmung der Kompetenzen als Pflegekraft von Arztseite
