# Supplementary material for: Long-Term Adoption of Televisits in Nursing Homes During the COVID-19 Crisis and Following Up Into the Postpandemic Setting: Mixed Methods Study
Source: JMIR Aging. 2024 Jun 6;7:e55471. doi: 10.2196/55471 (PMC11190630; doi:10.2196/55471)
Supplement: Multimedia Appendix 6 [file aging_v7i1e55471_app6.pdf]

Multimedia Appendix 6. Comments of the nurses regarding improvement potential for further televisits. The number of times a specific topic was given is indicated in brackets.

	Answers
**Theme: Training**	
	Need for more practical training (6)
	Need for more theoretical training (1)
	Difficulties to adapt to the technology after a long time without televisits (1)
**Theme: Technology**	
	Unstable internet connection (1)
	Bug with the camera (1)
	A portable camera would be better than the fixed one (2)
	Difficulties with the audio connection (1)
	The telemedical system should be linked to the care documentation program (1)
**Theme: Organization**	
	More availability of the physician needed (1)
	Improve information of the residents (1)
