# Supplementary figures and images for: Long-Term Adoption of Televisits in Nursing Homes During the COVID-19 Crisis and Following Up Into the Postpandemic Setting: Mixed Methods Study
Source: JMIR Aging. 2024 Jun 6;7:e55471. doi: 10.2196/55471 (PMC11190630; doi:10.2196/55471)

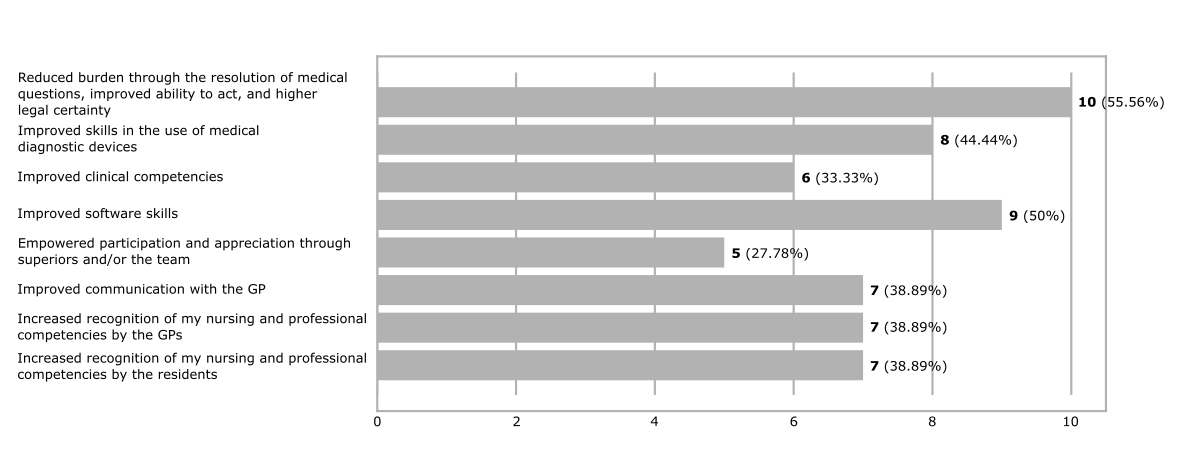

Supplement: Multimedia Appendix 7 [file aging_v7i1e55471_app7.png]
